# Evaluation of the Efficacy and Safety of Silver Nanoparticles in the Treatment of Non-Neurological and Neurological Distemper in Dogs: A Randomized Clinical Trial

**DOI:** 10.3390/v14112329

**Published:** 2022-10-24

**Authors:** Fabian Gastelum-Leyva, Antonio Pena-Jasso, Martha Alvarado-Vera, Ismael Plascencia-López, Leslie Patrón-Romero, Verónica Loera-Castañeda, Jesús Alonso Gándara-Mireles, Ismael Lares-Asseff, María Ángeles Leal-Ávila, J. A. Alvelais-Palacios, Javier Almeida-Pérez, Nina Bogdanchikova, Alexey Pestryakov, Horacio Almanza-Reyes

**Affiliations:** 1 Veterinary Clinic La Trova Zoo, Tijuana 22207, Mexico; 2 Pena-Jasso Veterinary Hospital, Ensenada 22880, Mexico; 3 Cluster de Bioeconomía de Baja California, A.C., Tijuana 22040, Mexico; 4 Faculty of Accounting and Administration, Autonomous University of Baja California, Tijuana 22390, Mexico; 5 Faculty of Medicine and Psychology, Autonomous University of Baja California, Tijuana 22390, Mexico; 6 National Polytechnic Institute, CIIDIR-Durango Unit, Durango 34220, Mexico; 7 School of Heath Sciences, Valle de Las Palmas, Autonomous University of Baja California, Tijuana 22260, Mexico; 8 Veterinary Clinic Jardines, Celaya 38080, Mexico; 9 Center of Nanoscience and Nanotechnology, UNAM (CNyN-UNAM), Ensenada 22860, Mexico; 10 Research School of Chemistry and Applied Biomedical Sciences, Tomsk Polytechnic University, 634050 Tomsk, Russia

**Keywords:** silver nanoparticles, canine distemper, canine distemper virus, neurological distemper, infectious diseases

## Abstract

Canine distemper is caused by canine distemper virus (CDV), a multisystemic infectious disease with a high morbidity and mortality rate in dogs. Nanotechnology represents a development opportunity for new molecules with antiviral effects that may become effective treatments in veterinary medicine. This study evaluated the efficacy and safety of silver nanoparticles (AgNPs) in 207 CDV, naturally infected, mixed-breed dogs exhibiting clinical signs of the non-neurological and neurological phases of the disease. Group 1a included 52 dogs (experimental group) diagnosed with non-neurologic distemper treated with 3% oral and nasal AgNPs in addition to supportive therapy. Group 1b included 46 dogs (control group) diagnosed with non-neurological distemper treated with supportive therapy only. Group 2a included 58 dogs with clinical signs of neurological distemper treated with 3% oral and nasal AgNPs in addition to supportive therapy. Group 2b included 51 dogs (control group) diagnosed with clinical signs of neurological distemper treated with supportive therapy only. Efficacy was measured by the difference in survival rates: in Group 1a, the survival rate was 44/52 (84.6%), versus 7/46 in Group 1b (15.2%), while both showed clinical signs of non-neurological distemper. The survival rate of dogs with clinical signs of neurological distemper in Group 2a (38/58; 65.6%) was significantly higher than those in Control Group 2b (0/51; 0%). No adverse reactions were detected in experimental groups treated with AgNPs. AgNPs significantly improved survival in dogs with clinical signs of neurological and non-neurological distemper. The use of AgNPs in the treatment of neurological distemper led to a drastic increase in the proportion of dogs recovered without sequels compared to dogs treated without AgNPs. The evidence demonstrates that AgNP therapy can be considered as a targeted treatment in dogs severely affected by canine distemper virus.

## 1. Introduction

Canine distemper (CD) is a multisystemic infectious disease with high morbidity/mortality in dogs worldwide, especially in unvaccinated or incompletely vaccinated dogs, caused by canine distemper virus (CDV) [1]. CDV is a single-stranded (negative-sense) enveloped RNA virus of the family Paramyxoviridae and genus Morbillivirus [2]. CDV infection causes acute, subacute, or chronic clinical disease. In the acute phase, viruses can be found in animal secretions [3]; this phase is followed by a variety of clinical signs, including the onset of a mild disease, with nonspecific emaciation and apathy, partial anorexia, rash, initial transient fever, severe nasal and ocular discharge, conjunctivitis, corneal ulcers, and lymphopenia 4–7 days postinfection. Subsequently, gastrointestinal (diarrhea) and respiratory (pneumonia) signs may appear, which are often complicated by secondary bacterial infections and neurological disorders [4,5]. Neurological signs may include hyperesthesia, cervical rigidity, seizures, cerebellar and vestibular signs, paraparesis or tetraparesis with sensory ataxia, myoclonus, nystagmus, postural reaction deficits, and tetraparesis or plegia [4,5,6]. Moreover, immunosuppression is caused by viral replication, which spreads to the lymph nodes through the blood and is disseminated throughout the body, with an incubation period of from 1 to 4 weeks [7]. Clinical signs vary depending on the virulence of the viral strain, environmental conditions, host age and host’s immune response [8].

A strong immune response can eliminate the virus before it multiplies in the host cells and can minimize clinical signs to achieve complete recovery in the animal, whereas a weak or delayed response can lead to virus propagation and death [3,9]. The treatment of CDV infection is usually based on symptomatic and supportive therapy, as there is no specific antiviral drug available for therapeutic use against CDV infection in domestic dogs [10]. Traditional treatment consists of clearing nasal and ocular secretions, as well as fluid therapy for dogs with impaired hydration status. Antipyretics are required for temperatures above 40 °C. Dogs with respiratory signs and secondary bacterial bronchopneumonia should receive antibiotics. Vitamin B may counteract anorexia. Anticonvulsants should be given when seizures are present. Corticosteroids are used to treat the immunopathologic basis of neural lesions and due to their potential to combat cerebral oedema. The passive administration of canine hyperimmune serum may be beneficial in combating viraemia and possibly viral replication in extraneural tissues. Immune stimulants are also used, but are reported to work only in the early stages of the disease, prior to the onset of neurological distemper [11,12,13].

The prevention and control of the disease are possible through active immunization [14]. Despite the availability of effective vaccines, outbreaks of CD have been reported in immunized dog populations in different parts of the world [15,16]. To find a treatment for canine distemper, different molecules were evaluated in vitro to study their antiviral effects. Among the antiviral agents, ribavirin (RBV) was used in CDV-infected Vero cells, showing a decrease in viral RNA accumulation using quantitative real-time RT-PCR. Low concentrations of RBV were effective in inhibiting virus replication, as, after three sequential passages in Vero cell monolayers, the virus was no longer detected [17]. Studies by Lanave G et al. (2017) evaluated the in vitro antiviral efficacy of RBV and boceprevir, alone or in combination against CDV. In infections in Vero cells, it was observed that, when used separately, they caused a slight decrease in viral load, unlike the combination of both drugs [18]. In Vero cells, the efficacy of antiviral action against CDV with RBV, interferon-alpha (IFN-α), and combinations of RBV and IFN-α was evaluated. Inhibitory effects were demonstrated at the intracellular level of the two compounds. When combined, a significant extracellular inhibition was detected [19]. Wang et al. (2014) evaluated the anti-viral activity of the interferon-alpha (FeIFN-α) and feline omega (FeIFNω), respectively, measuring the ability to inhibit the cytopathic effects of the virus at the cellular level. The results showed that the antiviral activity of FeIFNω was higher than FeIFN-α. The viral activity was 160-fold and 4-fold higher against H9N2 influenza virus and CDV than FeIFN-α [20]. Xue et al. (2019) conducted a study to evaluate the action of Favipiravir (T-705) in CDV-infected Vero and DH82 cells. They demonstrated that T-705 significantly inhibited replication at different concentrations, ranging from 2441 μg/mL to 1250 μg/mL [21]. Carvalho et al. (2013) evaluated the in vitro antiviral activity of flavonoids quercetin, morin, rutin and hesperidin, and phenolic cinnamic, trans-cinnamic and ferulic acids, showing strong viral inhibition when added early in the infection [22].

In vivo studies performed by Liu P. C. et al. (2008) demonstrated improved survival in CDV-affected puppies with non-neurological signs when they received IgG antibodies produced in pigs against CDV [8]. Jianlou Zhang et al. (2021) recently demonstrated a very similar therapeutic effect after the inoculation of IgG produced in Shih-tzu dogs (homologous animals) and produced in donkeys (Dezhou Donkey) or heterologous antiserum against CDV [23]. Bogdanchikova et al. (2016) demonstrated that treatment with AgNPs in a small number of dogs previously presenting with non-neurological signs showed an increased rate of recovery; however, in dogs with neurological signs, treatment with AgNPs was not able to reverse the disease [24]. As mentioned above, different molecules were developed to evaluate their antiviral effects in in vitro and in vivo studies for the treatment of CDV infection; however, there is no effective and safe antiviral treatment for CDV-infected dogs at present. Innovative developments were observed within nanomedicine, specifically in the area of applied veterinary medicine [25], to explore the effect alternative therapeutic molecules against CDV; this double-blind, randomized clinical study shows the results of the treating dogs naturally infected with CDV presenting clinical signs of non-neurological and neurological distemper by applying a new formulation of AgNPs with a conjugation of polyvinylpyrrolidone (PVP) and hydrolyzed collagen (CH).

## 2. Materials and Methods

### 2.1. Good Animal Handling Practices

The study was conducted in accordance with good animal handling and practices, and approved by the ethics committee of the Faculty of Medicine and Psychology of the Autonomous University of Baja California and the research ethics committee of the Jardines Veterinary Clinic. The study is reported in accordance with the ARRIVE guidelines [26]. Consent was obtained from the owner of each dog to participate in the study and the study complies with the Declaration of Helsinki.

### 2.2. Objective

To evaluate the efficacy and safety of silver nanoparticles (AgNPs) in dogs naturally infected with CDV showing clinical signs of non-neurologic and neurologic stages of the disease.

### 2.3. Hypothesis

The administration of oral and nasal 3% AgNPs in dogs naturally infected with CDV will delay the onset of clinical signs and death associated with CDV.

### 2.4. Study Design

A prospective cohort study was conducted to evaluate the factors associated with disease progression and the effect of AgNPs on survival rates in dogs diagnosed with non-neurological and neurological distemper disease. A four-arm, double-blind, randomized, structured, multicenter clinical study (veterinarians and clients were unaware of the treatment group) was performed.

### 2.5. Sample Size

Considering two relevant studies, such as those mentioned above, conducted with antibodies against CDV infection [8,23] and mesenchymal stem cell therapy in CD [10,27], a sample size >200 dogs was initially determined as a basis for strong evidence. However, no formal statistical calculation was performed to calculate the samples per group, due to the absence of a phase I canine study examining the effects of AgNPs on the treatment of non-neurologic and neurologic distemper. Client-owned dogs that met the inclusion criteria were recruited and admitted from two clinical centers in Tijuana and Ensenada, Baja Cali-fornia, México. A total of 207 dogs were enrolled in the clinical study to determine the efficacy and safety of AgNPs in dogs naturally infected with CDV and showing clinical signs of non-neurological and neurological distemper. Group 1 included 52 dogs diagnosed with non-neurological distemper that were treated with AgNPs orally and nasally over a 15-day period (Group 1a) in addition to supportive therapy; as a control group, 46 dogs received supportive therapy alone (Group 1b). Group 2 included 58 dogs with clinical signs of neurological distemper that were treated with AgNPs administered orally and nasally for a period of from 25 to 30 days in addition to supportive therapy (Group 2a), and 51 dogs (control group) that received supportive therapy only (Group 2a).

### 2.6. Enrollment Criteria

Dogs were eligible to participate in the study if the owner signed the informed consent. To be eligible for inclusion, the dog had to show clinical signs of naturally infected non-neurological and neurological distemper, with a positive serological test by indirect immunofluorescent assay (IFA) and confirmation by reverse transcription polymerase chain reaction (RT-PCR). Subsequently, after clinical treatment, there was a clear improvement in clinical signs: initially fever, anorexia, depression, leukopenia, mild respiratory symptoms, gastrointestinal and neurological problems, such as myoclonus and seizures, as well as presenting a negative RT-PCR test. The dogs were evaluated before and during treatment to assess clinical manifestations for inclusion in the group of dogs with non-neurological and neurological distemper. The veterinarian completed a clinical history, analyzing breed, sex, age, weight and body temperature, as well as clinical examinations and laboratory tests. During the clinical interview, owners were questioned about history off feeding difficulties, hyporexia, anorexia, fever, diarrhea, dysphagia, vomit, depression, conjunctival and nasal congestion, ocular or nasal serous discharge, and corneal opacity. On auscultation, respiratory difficulty, bronchitis, bronchopneumonia complications, cough and the presence of abnormal sounds in the lung were evaluated; abdominal spasm malnutrition, the dog’s depressive or irritable state, complications of secondary bacterial infection, and the presence of skin, nose and paw pads’ hyperkeratosis were also evaluated. During the evaluation of neurological signs, depression was gradually and progressively evaluated, as well as myalgias, myoclonus and spontaneous eye movements, segmental or generalized contracture attacks, including mandibular contractions or jaw tics and excessive salivation caused by these last movements, muscular spasms that developed paralysis of the extremities and motor incoordination, and the period of convulsions that gave rise to the development of permanent epileptic symptoms, after which the animal remains deprived of movement, as if asleep, loses consciousness and later falls into a coma. The dogs were evaluated weekly by a veterinarian to further assess the evolution of these clinical signs.

### 2.7. Exclusion Criteria

The dogs were excluded from the study if the owner refused to sign the informed consent form, if the dog did not show clinical signs of non-neurological and neurological distemper or if a negative serological test was obtained by IFA and negative confirmation by RT-PCR. Dogs with a history of recent administration (within 30 days of planned enrollment) of any drug listed in Table 1 were excluded. It was suggested that these medications should be considered as potential rescue therapy at any moment after the application of AgNPs.

### 2.8. Randomization

Dogs were formally enrolled on day 0 and randomized to groups with an allocation ratio of 1:1 in blocks of 10 to maintain similar sample sizes in all four arms (treatment and control groups) [28]. The randomization sequence was generated by the principal investigator randomly using the SAS/STAT software. Each investigator was initially assigned 5 consecutive case numbers from a block of 10, ensuring that each investigator or veterinarian did not know how many cases assigned to each treatment and control were under their care. When a veterinarian recruited a new dog, that dog was assigned the next available case number and received the pre-assigned treatment. During the recruitment, some case numbers were reassigned among participating veterinarians to meet the recruitment goal. The Consolidated Standards of Reporting Trials (CONSORT) were used to assist in trial performance, randomization, and data reporting [29].

### 2.9. Blinding

Veterinarians, investigators, owners, study monitors, assistants, and statistical staff were blinded to the treatment assignment. The blinding code for the four study groups was in the hands of the principal investigator. Blinding was achieved by a separation of duties: a treatment administrator (that is, the dispenser) at each Veterinary Hospital was responsible for dispensing and the administration of AgNPs and supportive therapy depending on the group. Predefined procedures were in place to allow for individual cases to be unblinded by a medical emergency from distemper complications. Unblinding was achieved by contacting the principal investigator who had the randomization list. They could then break the treatment code and inform the investigator and responsible veterinarian of the treatment that the animal was receiving [30].

### 2.10. Trial Medication

The AgNP formulation Argovit-C from the Vector-Vita Ltd. Center of Investigation and Production, manufactured in Novosibirsk, Russia, was applied. The AgNP solution was used at a concentration of 3%, according to the following formula: 1.88 g/100 g of polyvinylpyrrolidone, 0.94 g/100 g of hydrolyzed collagen, 0.18 g/100 g of metallic silver and 97 mL of distilled water. Spherical AgNPs with average sizes between 10 and 35 nm were used.

### 2.11. Population Analyzed

Any dog with clinical signs and serologic diagnosed non-neurological and neurological CD was randomized and either received at least one dose of the AgNPs during the study (experimental group) or support therapy only (control group). All the dogs in the population-based study were confirmed to meet all inclusion criteria (and none of the exclusion criteria) and were included in the study protocol until one of the following occurred: the dog met the baseline evaluation criteria, the dog was withdrawn from the initial assessment due to the occurrence of an event that prevented continuation of the study, or the dog reached the end of the study.

### 2.12. Experimental Animals

A total of 207 naturally infected mixed breed dogs with clinical signs of non-neurological and neurological CD were included. Dog characteristics such as breed, sex, age, weight, and rectal temperature at inclusion interview were recorded. The dogs were recruited through talks and conferences at different veterinary schools in Baja, California, to be referred to the two Veterinary Hospitals at which the study was conducted to verify that they met the inclusion criteria.

### 2.13. Experimental Procedures

In this study, a total of 207 naturally infected, mixed-breed dogs with clinical signs of non-neurological and neurological distemper were included. The AgNPs were administered at a target dose of 3% (1.88 g/100 g of polyvinylpyrrolidone, 0.94 g/100 g of hydrolyzed collagen, 0.18 g/100 g of metallic silver, and 97 mL of distilled water). In Group 1a, 52 dogs (experimental group) diagnosed with non-neurological distemper were treated with a combination of oral 1.5 mL/kg and nasal 0.25 mL/kg (with instillations of from 0.5 to 1 mL per nostril at intervals of 5–10 min until the dose was completed) every 8 h during the first 7 days. From day 8 to day 15, depending on the patient’s response, the dose was changed to oral 1 mL/kg every 12 h and nasal administration was eliminated (Group 1a), in addition to supportive therapy. A total of 46 dogs (control group) diagnosed with non-neurological distemper that only received routine supportive therapy (Group 2b) (Table S1) were included. In Group 2a, 58 dogs with clinical signs of neurological distemper were treated with a combination of oral 1.5 mL/kg and nasal 0.5 mL/kg (with instillations of from 0.5 to 1 mL per nostril at intervals of 5–10 min until the dose was completed) every 8 h during the first 7 days. In patients weighing 20 kg or more, the standard nasal dose of 10 mL was maintained. From day 8 to day 15, depending on the patient’s response, the dose was changed to oral 1.5 mL/kg every 8 h and nasal 0.25 mL/kg every 8 h (by drip at intervals of 5 to 10 min). In patients weighing 20 kg or more, the standard nasal dose of 5 mL was maintained. In this same Group 2a, from day 16 to day 30, depending on the patient’s response, the dose was changed to oral 1 mL/kg and the nasal administration, and the application of supportive therapy was eliminated (Group 2a). A total of 51 dogs (control group) diagnosed with clinical signs of neurological distemper that only received supportive treatment were included (Group 2b) (Table S1).

### 2.14. Concomitant Treatments

All concomitant medications and supportive therapy that dogs diagnosed with non-neurological and neurological distemper were receiving at enrollment and during the course of the study were recorded. Several dogs were excluded from the study if a veterinary physician identified the administration of antiviral drugs, interferons, immunotherapy, and investigational therapies. A list of such medications was prospectively defined and included in the protocol (Table 1). The medications whose administration did not cause the dogs to be excluded based on the veterinarian’s analysis are listed in Table S1. Supportive therapy was adapted to each individual animal based on its clinical signs on non-neurological and neurological distemper. Secondary bacterial infections were monitored, and the observed clinical signs were treated [31]. Respiratory symptoms were treated with preventive parenteral antibiotics with amoxicillin-clavulanate to avoid pulmonary complications associated with infections by opportunistic pathogens such as *Bordetella bronchiseptica* [32,33]. Regarding nasal and ocular exudates, antibiotics were applied in cases of bacterial infections; mucolytics and nebulizations were also administered in case they were required. Dogs that presented with gastrointestinal problems and dehydration, caused by digestive signs (vomiting, diarrhea) or anorexia, present in almost all sick animals, were intravenously administered with balanced electrolyte solutions. Ringer’s lactate solution was applied intravenously or subcutaneously, depending on the dog’s state of dehydration. Antiemetics were also administered if necessary, along with the solution [32,33,34]. Alterations such as hypoglycemia were controlled with glucose supplements. The use of antioxidants, vitamin E, B complex vitamins and high doses of vitamin A corresponded to essential therapeutic measures, to replace the vitamins lost due to anorexia and diuresis, or to stimulate appetite [32,33,35]. Antipyretics were used in dogs with temperatures above 40 °C. The use of anticonvulsant and sedative medication such as phenobarbital was indicated after the onset of systemic illness and before the onset of neurological signs [36,37]. To control optic neuritis, its sequelae of blindness, and relief signs of cerebral edema, steroid anti-inflammatory drugs were administered [38]. The treatment of neurological disorders such as multifocal encephalitis is progressive and, in some cases, led to tetraplegia and incapacitation, for which euthanasia was frequently indicated. Euthanasia was not used unless the neurological signs were progressive or incompatible with life, and was used with prior authorization from the owner [32,33,34]. Table S1 shows the medications used as support therapy in the clinical picture of non-neurological and neurological distemper [32,33,35].

### 2.15. Schedule of Events

Prior to inclusion, a medical history was completed from each dog; they then underwent physical examinations. Dog characteristics such as breed, sex, age, weight, and rectal temperature were recorded. Blood samples were collected on days 0, 14, and 28 by venipuncture, and complete blood counts and total leukocyte differential counts were determined using hematology equipment (Celltac α Automated Hematology Analyzer MEK-6510, Nihon Kohden, Tokyo, Japan), (Table 2). The blood chemistry panel included glucose, BUN, creatinine, cholesterol, triglycerides, total protein, albumin, globulin, ALP, ALT, AST, GGT, bilirubin, uric acid, phosphorus and amylase, determined with an automatic biochemical analyzer (Clinical Chemistry Analyzer, ILab 300 plus, Monsano, Italy), (Table 3).

### 2.16. Rescue Therapy

Interventional therapy (“rescue therapy”) was administered at any time the veterinarian determined that a dog was excessively uncomfortable or in pain due to complications with non-neurological and neurological distemper. The intervention treatment could include any medication (except those in Table 1) to control pain (Table S1). Euthanasia was not used unless the neurological signs were progressive or incompatible with life [32,33,34].

### 2.17. Premature Completion and Follow-Up

The dogs that were withdrawn from the study received rescue therapy that included the anesthetic drugs allowed by the protocol, mainly fluids and intravenous or oral antibiotics, and anticonvulsants such as phenobarbital, pregabalin, levetiracetam and diazepam, with no differences between groups (Table S1). These were received at any time at the discretion of the veterinarian. The dogs that received the treatment as the experimental group (Group 1a and Group 2a), were kept under observation in the different veterinary hospitals after the intervention and the possible adverse effects (AE) were documented. Owners of the dogs included in the study received periodic follow-up phone calls at approximately 3, 15, 45, 60, and 90 days after normal or premature termination to assess the animal’s general well-being.

### 2.18. Monitoring of Adverse Effects

A complete blood count, liver profile, total protein, and enzymes such as amylase and lipase were performed every two weeks during the study. Study participants were thoroughly trained by veterinarians to monitor the adverse events (AE) produced by the experimental drug and instructed to contact investigators if these were observed.

### 2.19. Efficacy Assessments

All study collaborators were notified that the same veterinarian should perform all efficacy evaluations for all cases at each site (experimental groups and control groups) whenever possible. The veterinarians from the different hospitals were trained to standardize the methods and measure the efficacy before the initiation of the study, in order to obtain the same evaluation criteria. The primary efficacy variable was treatment failure, which was defined by the occurrence of the following:

1. The need for rescue therapy to control complications of non-neurological or neurological distemper. Rescue therapy was administered any time the veterinarian determined that a dog was excessively uncomfortable or in pain.

2. Document the number of dogs that were prematurely withdrawn from the study due to adverse effects related to the experimental group.

3. Evaluate the progression of non-neurological and neurological distemper disease in the experimental and control groups after the assessment of clinical signs and sequelae presented by the dogs.

4. Document the percentage of dogs in the experimental and control groups for which euthanasia was indicated (when the owners reached consensus with the veterinarians) due to complications of non-neurological or neurological distemper.

5. Document the percentage of dogs in the experimental and control groups that presented with sudden death due to complications of non-neurological or neurological distemper.

### 2.20. Safety Assessments

Safety was analyzed in all the dogs that received at least one dose of AgNPs (experimental groups) or that only received support therapy (control groups). Data for the safety assessment included the documented AE, veterinary physician’s follow-up findings, clinical laboratory variables (blood chemistry test, liver profile, enzymes such as amylase and lipase, and complete blood count) collected before, during and at the end of treatment during the study.

### 2.21. Indirect Immunofluorescence

An indirect immunofluorescent assay (IFA) test was used for the determination of the serological test and the quantification of the IgM antibody titer against CDV using the Canine Distemper Fuller Commercial Kit and an origin canine serum that was positive for antibodies against CDV, using the available protocol according to the manufacturer’s instructions.

### 2.22. Hemi-Nested RT-PCR Assay for CDV Detection

On the first day (day 0), blood samples, urine, nasal, rectal and oral swabs were collected in a 1 mL phosphate-buffered saline solution. Viral RNA was extracted from 300 µL of the mixture using the TRIzol^®^ (Life Technologies, EE. UU.) method, according to the manufacturer’s instructions. The extracted RNA was used to perform the first reverse transcription-polymerase chain reaction (RT-PCR). The primers used were those described by Di Francesco et al. (2012), with modifications to the protocol [39]. These primers are specific to a 350 base-pair (bp) fragment, within the conserved part of the *NP* gene of the CDV genome for the first RT-PCR amplification. Reverse transcription was carried out with the *SuperScript III* (Invitrogen) enzyme and the conditions recommended by the manufacturer. First, the template RNA (1 μg) was incubated with the oligonucleotide REV1441 and with dNTPs (1 mM final), in a volume of 10 μL, for 5 min at 85 °C, and then quickly transferred to ice. Next, the remaining components of the reaction (reaction buffer, DTT, MgCl2, RNAse inhibitor and *Superscript III* enzyme) were added at a final volume of 20 µL, and incubated for 1 h at 50 °C, followed by 5 min at 85 °C. Subsequently, 5 µL of the RT reaction was used as a template to amplify by PCR using primers C2 and REV1441, and 35 cycles of denaturation at 94 °C for 30 s, primer hybridization at 48 °C for 45 s, extension at 72 °C for 45 s, and final extension at 72 °C for 7 min.

The second amplification was carri”d ou’ with primers FW1261 and REV1441, specific to the internal 180 bp fragment of the first amplicon, using the enzyme Taq polymerase enzyme (Roche) and the manufacturer’s mixture. The reaction mixture was incubated at 95 °C for 2 min and the amplification was obtained through 30 cycles of denaturation at 95 °C for 30 s, primer hybridization at 48 °C for 45 s, extension at 72 °C for 45 s, and final extension at 72 °C for 7 min. The thermocycler was the Applied Biosystems 2720 Thermal Cycler.

The amplicons were analyzed in 1% agarose gel, and the electrophoresis results were observed under a UV illuminator after ethidium bromide staining, confirming the amplification with a 180 bp fragment [39].

### 2.23. Statistical Analysis

Statistical analyses were performed using SPSS Statistics 28.0. Continuous variables (age, weight, IgM value, temperature) were expressed as the mean and standard error (SE). All continuous data were assessed utilizing a Shapiro–Wilk test and D’Agostino-Pearson for normality. The differences in the survival probability rates between the groups were evaluated using Kaplan–Meier curves and Log-rank 1, Log-rank 2, Wilcoxon and Tarone–Ware test. In the statistical analyses, *p* < 0.05 was considered significant

## 3. Results

### 3.1. Study Population

A total of 207 naturally infected, mixed-breed dogs with clinical signs of non-neurological and neurological distemper were included. Four groups were included in this study. Group 1a included 52 dogs with non-neurological distemper treated with AgNPs (Table S2). The mean age was 42.88 months; the mean body weight was 15.39 kg; the mean survival was 15.39 days; the average temperature was 40.02 °C; the mean IgM was 1: 146; 29 were males and 23 were females. In this group of 52 dogs, 44 survived and 8 canines died, which represents 84.6% survival and 15.2% mortality.

Group 1b included 46 dogs as a control group that only received supportive therapy (Table S3). The mean age was 56.13 months; the mean body weight was 17.75 kg; the mean survival was 8.78 days; the average temperature was 39.88 °C; the mean IgM was 1: 128; 23 were males and 23 were females. In this group of 46 dogs, 7 survived and 39 canines died, which represents 15.2% survival and 84.8% mortality.

Two more groups of dogs with clinical signs of neurological distemper were included. Group 2a included 58 dogs with clinical signs of neurological distemper (Table S4), that were treated with a combination of oral 1.5 mL/kg and nasal 0.5 mL/kg of 3% AgNPs every 8 h during the first 7 days and modification of these doses depending on the patient’s response for up to 30 days, in addition to the application of routine supportive therapy. The mean age was 48.28 months; the mean body weight was 16.61 kg; the mean survival was 22.5 days; the average temperature was 40.21 °C; the mean IgM was 1: 160; 32 were males and 26 were females. In this group of 58 dogs, 38 survived and 20 canines died, which represents 65.6% survival and 34.4% mortality.

Group 2b included 51 dogs as a control group that only received supportive therapy (Table S5). The mean age was 51.4 months; the mean body weight was 16.04 kg; the mean survival was 11.94 days; the average temperature was 40.19 °C; the mean IgM was 1: 117; 24 were males and 27 were females. In this group of 51 dogs, all the canines died, which represents 0% survival and 100% mortality.

### 3.2. Monitoring of Adverse Effects

In Group 1a with diagnosis of non-neurological distemper, two dogs were removed from the study after two days of treatment with oral and nasal AgNPs, because they presented diarrhea, vomiting and fever that reached 40 °C in both cases. The treatment was paused, the dogs recovered with supportive therapy for 3 days and, in 24 h, they were included in the protocol again. In addition to these two dogs, there was no other clinically significant adverse effect after the administration of AgNPs, except for the elevation of hepatic levels over a period of time in some dogs. A mixed linear model was performed to measure the repeat values of glucose, BUN, globulin, ALP, ALT, AST, GGT, total bilirubin, uric acid, phosphorous, amylase, lipase, so that possible hepatic alterations could be evaluated. Serum biochemical analysis were performed at different times, and it was determined that the pre-treatment values were not significantly different between the two groups except for alkaline phosphatase (ALP), alanine aminotransferase (ALT) and aspartate aminotransferase (AST), which significantly increased over time from the start of treatment. However, the different values remained within the reference ranges. The values of the complete blood count are not presented since the results were not significant (Table 3).

### 3.3. Adverse Reactions after Administration of AgNPs

No adverse reactions were reported after the administration of AgNPs in the two groups that were administered with oral and nasal AgNPs (Groups 1a and 2a).

### 3.4. Rescue Therapy and Concomitant Treatments

Even though the veterinarians were authorized to proportionate rescue therapy at any moment after the application of AgNPs, none of the dogs received the therapy or medications listed in Table 1. The only concomitant treatment or support therapy included the anesthetic drugs permitted in the protocol, mainly IV or oral fluids and antibiotics, anticonvulsants such as phenobarbital, pregabalin, levetiracetam and diazepam, without any differences between the groups (Table S1).

### 3.5. Immune Response

In Group 1a, the mean IgM value was 1:146, considering only the dogs recovered without sequelae, the average IgM was 1:154, of those recovered with sequelae was 1:144 and of the deceased 1:110. The mean IgM value for Group 1b was 1:128, considering only the dogs recovered without sequelae, the mean IgM value was 1: 280, of those recovered with sequelae was 1:240 and of the deceased 1:105. The mean IgM value for the total of Group 2a was 1: 137, considering only the dogs recovered without sequelae, the mean IgM value was 1: 160, of those recovered with sequelae and of the deceased 1:116. The mean IgM value for the Group 2b was 1:117, in this group all died. The relationships presented in these studies corroborate the relationship between survival to the disease and the response of the immune system of dogs.

### 3.6. Survival

The results presented show a clear difference between the number of dogs that survived canine distemper disease in the groups in which AgNPs were administered (1a and 2a) compared with those that only received supportive therapy (1b and 2b). This was true for both the groups of non-neurological CD (1a and 1b) and the groups diagnosed with neurological distemper (2a and 2b), significantly highlighting the efficacy of the treatment with AgNPs (Table 4). Survival without sequels in Group 1a (non-neurological) was 7.8 times higher than those with sequels, while in the control group 1b, those that recovered without sequels were 0.75 fewer than those with sequels. Hence, treatment with AgNPs in the non-neurological group 1a increased the proportion of dogs that recovered without sequelae compared with dogs in the control group 1b (Table 4).

The Kaplan–Meier procedure in SPSS version 28.0 was used to statistically measure the difference in the probability of survival between the experimental and control groups, as well as to observe the time period in which the events occurred—in this case, the death of dogs in the clinical study. First, we performed the tests with the Log Rank, Breslow and Tarone–Ware methods based on the Chi-Square statistic to demonstrate that there is a statistically significant difference between the survival results of groups 1a and 1b (Table 5), and 2a and 2b (Table 6). This test compares expected and observed events at each event time in both groups. If we reject the null hypothesis and find that groups have a significant difference in survival rates, then, comparing the experimental group to the control group, there is evidence of the effect of AgNPs.

We performed the Kaplan–Meier test on the groups to determine the differences in survival rates. The rates were the same in groups 1a and 1b, with 84.5% versus 15.2%, and groups 2a and 2b, with 65.5% versus 0.0% (Table 7 and Table 8).

The average overall survival for groups 1a and 1b was 11.4 days and, when separated by group, the average survival for the experimental group was 13.8 days versus 8.8 days for the control group. A 56% greater difference was observed in the experimental group versus the control group (Table 9). The analysis of groups 2a and 2b showed that the average survival for the whole was 17.6 days and, when separated by groups, the average survival for the experimental group (2a) was 22.5 days, versus 11.9 days for the control group (2b). An 89% greater difference was observed in the experimental group versus the control group (Table 10).

**Figure 1:**
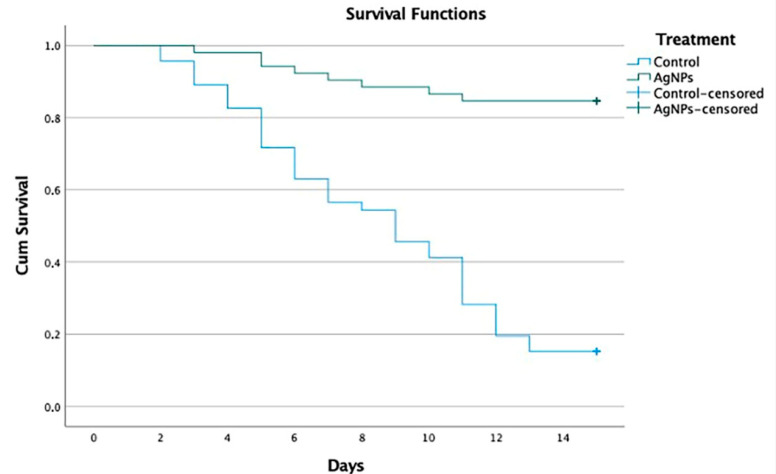
Comparison of survival rates between Groups 1a and 1b. In Group 1a (experimental), the probability of survival was maintained at above 85%, whereas in group 1b (control), this curve declined on day 13 down to 15%.

**Figure 2:**
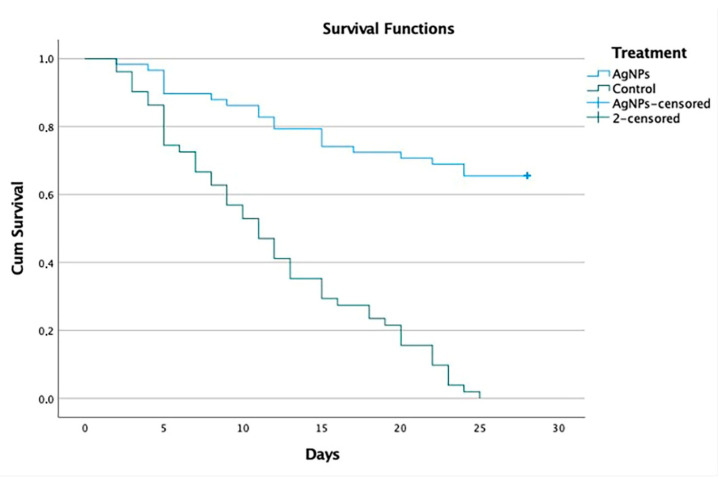
Comparison of survival rates between Groups 2a and 2b. In group 2b (experimental), the survival rate was maintained at above 65%, whereas in group 2b (control), the curve considerably declined, with a survival rate of 0% on day 25.

The Kaplan–Meier survival curves represent the estimated survival percentage of dogs in groups 1a and 1b (Figure 1) and 2a and 2b (Figure 2) who met the inclusion criteria (clinical signs of non-neurological distemper), in accordance with study time. Figure 1 presents a comparison of survival rates between Groups 1a and 1b. The green line represents the group that received AgNP treatment, and the blue line represents those that received supportive therapy. The experiment was limited to 15 days’ treatment, so at the end of both lines, we can see a vertical line showing an 85% survival rate for the dogs in the experimental group versus 15% for dogs in the control group.

Figure 2 presents a comparison of the survival rates between Groups 2a and 2b. The blue line represents the group that received AgNP treatment, and the green line represents those that received supportive therapy. The experiment was limited to 28 days’ treatment, so, at the end of both lines, we can see a vertical line, with the blue line showing a survival rate of 65% for dogs in the experimental group versus 0.0% for dogs in the control group.

Finally, to calculate the effect size, we measured the odds ratio. If we look at our sample, we can first calculate the odds that a dog survives following AgNP treatment in groups 1a and 1b.

OddsGroup1a = 44 survived/8 died = 5.5

OddsGroup1b = 7 survived/39 died = 0.18

Odds ratio = OddsGroup1a/OddsGroup1b = 5.5/0.18 = 30.5

This tells us that if a dog received AgNP treatment, the odds of survival were 30.5 times higher than if the dog only received supportive therapy. In the neurological distemper groups (2a and 2b), no dogs in the control group survived; for this reason, odds ratios were not calculated.

## 4. Discussion

Canine distemper is a potentially serious, multi-system, infectious disease characterized by a high morbidity/mortality in dogs [1]. Supportive and symptomatic therapies are the current guidelines for the treatment of affected dogs. Despite the availability of effective vaccines, outbreaks of CDV have been recorded in different parts of the world in immunized dog populations [1,15,16,17]. Outbreaks of CDV have also been reported in animals ranging from stray dogs to zoo animals such as wild tigers and giant pandas, highlighting the zoonotic and spillover characteristics of CDV [40]. Some antiviral drugs have been reported to have an effect in vitro, but none of them have demonstrated clinical efficacy in dogs diagnosed with non-neurological and neurological distemper [18,19,20,21,22,23]. The development of new antiviral drugs based on nanotechnology could offer a promising short-term option. The application of AgNPs as an antiviral agent provides an alternative treatment for CDV-infected dogs. It has been proposed that AgNPs act by inhibiting viral infection in the *Paramyxoviridae* family, particularly during the binding and entry of the different viruses of this family into the cell.

This finding is consistent with previous studies that observed associations between the in vitro neutralization of AgNPs and the experiments performed by L. Sun et al. (2008), which demonstrated that AgNPs conjugated with polyvinylpyrrolidone (PVP) showed low toxicity to Hep-2 cells at low concentrations and exhibited 44% inhibition of the respiratory syncytial virus (RSV) [41]. Morris D. et al. (2019) demonstrated the reduction mediated by AgNPs in RSV replication, both in epithelial cell lines and in experimentally infected BALB/c mice, concluding that AgNPs manage to adhere to viral glycoproteins, blocking entry to the host cell [42]. Gaikwad S. et al. (2013) demonstrated that AgNPs experience a size-dependent interaction with the human parainfluenza virus type 3 (HPIV-3); AgNPs can reduce viral infectivity, probably by blocking the interaction between the virus and the cell [43]. Yin J. et al. (2013) conducted studies with MDCK cells in a murine model with HPIV, concluding that AgNPs had inhibitory effects in vitro and in vivo and their possible mechanism is the inhibition of neuraminidase and subsequent damage to the virion [44]. Khandelwal N. et al. (2014) evaluated the antiviral effect of AgNPs against Peste des Petits Ruminants virus (PPRV), a prototype *Morbillivirus*. Vero cells with AgNPs at a non-cytotoxic concentration significantly inhibited PPRV replication in vitro, exerting a blocking effect on PPRV entry to the target cell [45]. The veterinary pharmaceutical formulation of AgNPs stabilized with polyvinylpyrrolidone (PVP) and hydrolyzed collagen (CH) used in this work showed very low toxicity in C2C12 and L929 mouse fibroblast cells, as well as in different experimental models (Wistar rats and New Zealand rabbits). The modification of the AgNP stabilizer, in comparison with our previous results [24], allowed for us to describe a treatment that significantly increases survival in dogs with neurological distemper, which is the last stage of the disease and the most difficult to treat successfully.

### 4.1. Immunological Biomarkers

It is known that not all animals infected with CDV die with, this being dependent in many cases on the infecting viral variant and the effective protective immune response of the infected animal [46]. Depending on the degree and immunological recovery, animals may die rapidly or recover after developing mild or even subclinical disease [47,48]. In this study, we used the value of virus-specific immunoglobulin M (IgM) as a marker of recent CDV infection [49,50]. The average IgM for the total of Group 1a was 1:146; when considering only the recovered dogs without sequelae, the average IgM was 1:154, while with the deceased dogs the average was 1:110. In Group 1b, the average IgM was 1:128, selecting only the recovered dogs without sequelae, the average IgM was 1:280, and taking only the deceased dogs, the average was 1:105. In Group 2a, the average IgM was 1:137; selecting the recovered dogs without sequelae, the average was 1:160, while the average IgM of the deceased dogs was 1:116. The average value of IgM for the total of Group 2b was 1:117; in this group, all the dogs died. The IgM titers obtained in these studies corroborate the relationship between survival and the response of the dog’s immune system; this allows us for to suggest that the IgM with a higher titer offers a greater probability of recovery. In a study by Jozwik et al. (2004), nine dogs infected with CDV were included, as confirmed by immunofluorescence test or RT-PCR; five of these dogs had high anti-CDV antibody titers (160–1280), and all of these animals recovered. However, the remaining four dogs, with low anti-CDV antibody titers (5–40), died [51]. The results of Bogdanchikova et al. (2016) demonstrate higher values of anti-CDV IgM antibodies at the early stages of the disease (non-neurological), where the dogs recovered favorably with the AgNP treatment. For neurological stages in combination with decreased anti-CDV IgM antibodies, treatment is not effective. Only one case (1/15) presented with an IgM titer of 1:320 and neurological diagnosis, which recovered compared to the rest of the cases with an IgM below 1: 160, due to its stronger immune response, concomitantly with AgNP treatment [24]. In relation to the treatment of neurological distemper with the application of AgNPs and IgM titers, we were able to characterize those nine dogs (N-4, N-7, N-8, N-18, N-22, N-31, N-34, N-39 and N45) that presented an average titer of 1:70; all recovered without neurological sequelae (see Additional File 3). We observed that, during AgNP treatment in these dogs with signs of neurological distemper, if the dog did not lose its appetite, did not lose weight and continued to stay hydrated, there was a good enough prognosis to continue with the treatment, preventing dogs from being euthanized.

Some factors in our study could reflect the changes that occurred after AgNP treatment during the clinical course of the disease, since the prognoses associated with mortality in neurological stages were very heterogeneous (surviving without neurological sequelae with a low titer of 1:70 of IgM). In previous studies, a strong association was found between high titers of anti-CDV antibodies (IgM) and recovery of dogs and low titers of anti-CDV antibodies and death from CDV [50,51]. The prognostic factors identified in our study must be evaluated in a comprehensive manner and not in isolation. These prognostic factors of improvement in treatment include adequate hydration for the dog, a balanced diet, supportive therapy (Table S1) and the correct application of oral and nasal AgNPs. Although nine dogs diagnosed with neurological distemper showed low IgM titers, they survived the disease. It should be noted that different authors have published on the application of AgNPs as neutralizers against viruses of the *Paramyxoviridae* family [45,46,47,48,49]. The neutralizing activity of AgNPs (Argovit-C) has been characterized in vitro and in vivo against different viruses in preclinical studies with Rift Valley fever virus [52], in infectious bronchitis coronavirus in chickens [53] and, recently, in human clinical studies against the SARS-CoV-2 virus [54]. This antiviral capacity is the main prognostic factor that must be considered to evaluate the clinical course of the disease and facilitate decision-making, as well as provide advice to veterinarians and guardians, when handling dogs diagnosed with neurological distemper.

### 4.2. Treatment

The present work is a continuation and extension of our previous work [24] and is dedicated to the application of different formulations of AgNPs for the treatment of CD. The differences between these studies are as follows:

1. The previous work included three groups with a small number of 40 dogs [24], while the present work was designed as one of the largest prospective, randomized, double-blind, multicenter, four-arm clinical studies in veterinary medicine involving 207 naturally infected dogs.

II. In the previous work, only AgNPs conjugated with low-molecular-weight PVP (3.6 Kda) were applied [24], in comparison with the mixture of two stabilizers (PVP of average molecular weight 12.5 kDa and CH) in this study.

III. In the previous work, the AgNPs presented an average particle size of from 0.5 to 100 nm [24]; in this study, spherical AgNPs with average sizes between 10 and 35 nm are presented.

Due to the improvements described earlier in the present work, the recovery of dogs with neurological symptoms increased from 6.6% (obtained in the previous work) [24] to 65.5%. Therefore, the modifications in the conjugation and the particle size of the AgNPs had an improvement in the treatment of neurological distemper.

The main objective of this study was to evaluate the efficacy and safety of AgNPs in dogs naturally infected with CDV that showed clinical signs of non-neurological and neurological stages of the disease. To evaluate the efficacy of the drug, all study collaborators were specified that the same veterinarian should perform all the efficacy evaluations for all the cases at each site (experimental groups and control groups), whenever possible. The interpretation of these results assumes that the measurements are stable, which could be the case in patients with clinical signs of non-neurological and neurological distemper. Efficacy assessment was determined by treatment failure, which was defined by the occurrence of:

2. The need for rescue therapy: the veterinarians were authorized to provide rescue therapy at any moment after the application of AgNPs, but none of the dogs within the protocol received rescue therapy (Table 1). Most of them only received supportive therapies, including the anesthetic drugs allowed in the protocol, mainly fluids and intravenous or oral antibiotics, and anticonvulsants such as phenobarbital, pregabalin, levetiracetam and diazepam, with no differences between groups (Table S1).
3. Premature withdrawal due to adverse effects: there were no significant differences in the frequency of reported adverse events in the two groups in which AgNPs were applied; this treatment was well-tolerated in both groups in this study. Although adverse effects were reported, most of them were mild or benign and classified as unrelated to the treatment. Only two dogs diagnosed with neurological distemper (Group 2a) were withdrawn from the study after two days of treatment with oral and nasal AgNPs, since they presented liquid diarrhea, vomiting and a temperature that reached 40.0 °C in both cases. Treatment was stopped and the dogs recovered on supportive therapy for 3 days and in 24 h they were again included in the AgNP treatment protocol. Apart from these two dogs, no other clinically significant adverse effects were observed following the administration of AgNPs, except for a slight increase in liver parameters over time in some dogs that never exceeded normal values. These slight changes were attributed to the combination of AgNPs and supportive therapy during treatment. The serum biochemical evaluation profile provides supporting evidence for the toxicity and safety of treatment with AgNPs. These findings indicate that the chronic administration of AgNPs in this population is safe.
4. Evaluate the disease progression after the assessment of clinical signs and sequelae presented by the dogs: the results referring to the clinical signs show that all animals presented with one or more systemic signs of disease caused by CDV. Most of these occurred before they presented neurological signs, after recovery from respiratory and gastrointestinal clinical signs. The neurological manifestations of CDV and its clinical expressions can vary due to the CNS being involved with the white matter as well as the gray matter [46]. A wide variety of neurological clinical signs (abnormal behavior, ataxia, paralysis, spasms, tics, prostration, paresis, cachexia, coma, myoclonus and convulsions) can be observed in the animal and a high percentage of patients are killed or euthanized due to poor disease prognosis [55]. In Group 1a, 44 of 52 (84.6%) dogs survived, 39 (75.0%) without sequelae and 5 (9.6%) with sequelae, whereas 7 of 46 (15.2%) dogs survived in Group 1b, 3 (6.5%) without sequelae and 4 (8.7%) with sequelae (Table S5, Figure 1). In Group 2a, 38 of 58 (65.5%) dogs survived, 28 (48.3%) without sequelae and 10 (17.2%) with sequelae, whereas 0 of 51 (0%) dogs survived in Group 2b (Table S5, Figure 2). In the experimental group 1a, five dogs were reported to survive with mild neurological sequelae, such as slight myoclonus or jaw tics, in addition to digestive, respiratory, and ophthalmological signs. In control group 1b, four dogs recovered with neurological sequelae, being mainly parietal, and jaw tics, myoclonus, prostration, and ataxia. In group 2a, 10 dogs presented signs of neurological lesions, such as myoclonus, ataxia and jaw and parietal tics, seizures, and claudication. In group 2b, where all dogs died, the neurological signs were parietal and jaw tics, myoclonus, seizures, paralysis of the swallowing reflex, spasm, prostration, coma, and ataxia. Regarding the onset of neurological signs, our results correspond to those reported by Koutinas et al. (2002); in most of our cases, these were gradual onset with a progressive chronic course, and in those animals that died were progressive and multifocal, the predominant neurological sign was the presence of myoclonus, both localized and generalized, in 13 of the 19 dogs [56]. Regarding the percentage of clinical signs and sequelae presented by the dogs with respect to age, our results coincided with a study carried out in Brazil, which included 70 dogs aged between 2 months and 13 years; in almost half (33; 47.1%), the dogs were up to one year of age, and in about a third (24; 34.3%) of the cases, the dogs were up to six months. Thirty cases (42.8%) occurred in dogs that were between one and six years of age; only seven (10.0%) cases occurred in dogs that were seven years of age or older [57]. These results are similar to those of a North American study that collected data on more than 100 cases of distemper with neurological manifestations, noting that just over half of the cases occurred in dogs up to one year of age, approximately one third of these dogs were less than four months old and most cases occurred in adult dogs [12].
5. Percentage of dogs for which euthanasia was indicated: in Group 1a, 8 of 52 dogs (15.38%) died, 7 (13.46%) due to the disease and 1 (1.92%) due to euthanasia; in Group 1b, 39 of 46 dogs died (84.78%), 31 (67.39%) died due to the disease and 8 (17.39%) due to euthanasia. In Group 2a, 20 of 58 dogs (34.48%) died, 12 (20.69%) due to the disease and 8 (13.79%) due to euthanasia; in group 2b, 51 of 51 dogs (100.0%) died, 36 (70.59%) due to the disease and 15 (29.41%) due to euthanasia. Regarding the percentage of dogs that were euthanized, our results correspond with those reported by Ranjithkumar et al. (2021), who recorded that the neurological forms of CDV were found in the group of 1–3 years of age. This coincides with the two proposed forms of disease progression, where the primary form states that the progress of cases of distemper encephalomyelitis was initially catarrhal signs, then the appearance of epilepsy, followed by paraplegia, and finally death/euthanasia. The second form presents epilepsy as an initial sign, which can progress to status epilepticus, and then to coma, and finally death/euthanasia [37].
6. Percentage of dogs that presented sudden death: regarding the percentage of dogs that presented spontaneous deaths, according to the diagnosis of non-neurological distemper and [38,41] neurological distemper, our results presented with mild manifestations of the disease, which varied from practically no clinical signs to severe effects, with a mortality rate between 50 and 100%, as reported by different studies [37,38,58,59,60,61]. The study shows, for the first time, convincing evidence of the benefit of treatment based on AgNPs in dogs naturally infected with CDV that showed clinical signs in non-neurological and neurological stages of the disease. Dogs that were administered with oral and nasal AgNPs showed a higher survival rate, reaching 84.6% in dogs with non-neurological distemper (Group 1a) and 65.5% in dogs with neurological distemper (Group 2a), delaying the appearance of clinical signs and spontaneous deaths related to distemper or euthanasia. Comparing these with the survival rates of control group 1b, at 15.2% with non-neurological distemper, and control group 2b, at 0% with neurological distemper, statistically demonstrates the difference between the survival rates. In addition, it was shown that, with a 15-day oral and nasal treatment of AgNPs in dogs that survived with a diagnosis of non-neurological distemper, 75% did not present neurological sequelae and only 9.6% survived with neurological sequelae. Likewise, it was shown that, with a 30-day oral treatment of AgNPs in dogs that survived with a diagnosis of neurological distemper, 48.3% did not present neurological sequelae and only 17.2% survived with neurological sequelae. The statistical analysis shows a significant difference at 99% reliability in the survival results obtained for both dogs with non-neurological distemper and, most importantly, dogs with neurological distemper. The survival rate decreases from 85 to 65% when compared to the control group, to 15 and 0%, respectively. These differences are relevant, and we can conclude that both neurological and non-neurological distemper treatment with AgNPs are effective.

Studies by Liu PC et al. (2008) recruited naturally infected puppies (six months of age) with severe respiratory signs but without neurological signs, which included 25 dogs (Group 1) that were treated with IgG produced in pigs against CVD and supportive therapy and 16 puppies receiving only supportive therapy (Group 2). They presented a significantly higher survival rate for dogs from Group 1 (19/25; 76%) than dogs in Group 2 (5/16; 31.3%) [8]. During therapy, 8 of 25 dogs (32%) in Group 1 developed neurological signs, versus 12 of 16 dogs (75%) in Group 2. In the studies conducted by Jianlou Zhang et al. (2021), 36 dogs, divided into three groups of 12 dogs, were recruited and infected with CDV (3 mL of 10 × 6 TCID 50) and protected with IgG produced in Shih-tzu dog and donkeys. Groups of dogs were classified into CDV + donkey IgG, CDV + dog IgG and CDV without IgG administration. The survival rate of dogs infected with CDV and without the IgG administration was 25% (3/12); however, there was no significant difference in the survival rate of dogs between the CDV + donkey IgG group (75%, 9/12) and the CDV + dog IgG group (83%, 10/12) [23]. Previous studies only evaluated the effect with homologous and heterologous IgG in early stages of the disease, without any dog being diagnosed with neurological distemper. Therefore, this is the first study to show AgNPs with a protective effect in dogs with clinical signs and a diagnosis of neurological distemper.

### 4.3. General Strengths and Limitations

The trial has methodological strengths and limitations that should be taken into account when interpreting the results of the study.

#### 4.3.1. Strengths

The main strength of the trial is its design: it is the largest prospective, randomized, double-blind, multicenter, four-arm clinical study in veterinary medicine, involving 207 naturally infected mixed-breed dogs with clinical signs of non-neurological and neurological distemper, within the range of similar studies of human patients [62,63]. All inclusion and exclusion criteria for study animals were independently verified by an evaluation criteria committee. In addition, the breed, body weight, and age distributions of the dogs in our study were similar to those reported in previous studies that recorded dogs diagnosed with non-neurological and neurological distemper [8,10,23]. The comparison of control group and placebo group allows for the identification of possible effects of the intervention medication (AgNPs) that go beyond the normal recovery capacity of the patients, which is known to be low in patients diagnosed with neurological distemper [24]. The blinding of participants, separation of duties and designation of a treatment administrator (that is, the dispenser), data analysis, outcome assessment and efficacy assessment greatly reduce the risk of bias in the current trial, supporting the internal validity of the study findings.

There were no significant differences between the groups of dogs where AgNPs were applied (group 1a and group 2a) and the possible adverse events that were observed, indicating that the administration of AgNPs by oral and nasal routes is safe and well tolerated. This is despite the fact that dogs in group 2a spent more time in the study and were, therefore, at risk of experiencing adverse events for a longer period. This is the first clinical trial where a clearly effective treatment for neurological distemper is reported, having shown convincing evidence of efficacy where the proliferation of the disease, spontaneous deaths and euthanasia were significantly decreased. Another strength of the study is the absence of the confounding effect of any antiviral or immunomodulatory medication; this restriction led to the need to withdraw several dogs as they did not meet the inclusion criteria (Table 1). An additional strength of the study is that, to our knowledge, there are no published data on the efficacy of a single medication for the treatment of neurological distemper, as used in our study.

This clinical trial was mostly sponsored by a pharmaceutical company that develops AgNPs (Vector-Vita Ltd.). It also received a contribution from Russian funds from the Ministry of Education, with the collaboration of different public universities and the participation of a bioeconomy cluster from Tijuana Baja California, A.C., that linked the institutions; however, industry funding of clinical trials is recognized as a potential source of bias in the results [64,65,66]. It should be noted that randomized clinical trials in humans are the ones that are most sponsored by the pharmaceutical industry and indicated with the most bias [67,68,69,70,71]. In applied veterinary research, there are no public or independent institutions at the national level, such as the National Council for Science and Technology (CONACYT), or institutions with international financing, such as the National Institutes of Health (NIH), which can provide finance through for public projects using randomized clinical trials in veterinary medicine. Therefore, these projects can only be carried out with the support of the pharmaceutical industry. The study presents the results of the safety and efficacy of AgNPs for use in dogs diagnosed with non-neurological and neurological distemper, a condition for which there is currently no evidence of a benefit with other treatments. Publication bias was also minimized as the sponsoring company was not involved in the analysis and publication of the results.

#### 4.3.2. Limitations

Comparing groups in randomized trials is challenging, particularly when these trials are conducted with dogs diagnosed with non-neurological and neurological distemper. The rate at which individuals drop out of these groups is uneven, non-random, and potentially related to complications of distemper and the different variables that are being measured [72]. For example, in this randomized study, the group of dogs diagnosed with neurological distemper was more likely to drop out of the trial [29]. The diagnosis of non-neurological and neurological distemper and confirmation of death related to distemper complications remain challenging in clinical trials. In this study, we attempted to minimize the impact of distemper diagnosis by using a blinded consensus check independent of the inclusion criteria, since it is not possible to irrefutably confirm whether any death is related to distemper. In the case of dogs that died spontaneously, the investigator classified the death produced by the consequences of non-neurological and neurological distemper based on the circumstances of death and his own opinion. Post-mortem examinations were not performed systematically, so it is possible that, in some cases, death was attributed to a complication caused by distemper when, in fact, it was due to another condition (and vice versa). For this reason, there was no histopathological analysis to confirm the diagnosis of neurological and non-neurological distemper, that is, to categorize the main pathologies according to the macroscopic morphological and histopathological necropsy findings by groups that contemplate the different systems affected by distemper, especially when clinical information and epidemiological data are considered. To decrease this risk of misdiagnosis, several measures were taken:

- Clinical and epidemiological information for each dog was available to the reviewers.
- Clinical signs of naturally infected dogs with non-neurological and neurological distemper, with a positive serological test by Indirect Immunofluorescent Assay, were registered.
- To confirm an acute CDV infection, the virus-specific IgM titer was determined.
- Diagnosis was confirmed with Reverse Transcription Polymerase Chain Reaction (RT-PCR).
- Subsequently, after clinical treatment, an RT-PCR test was performed, resulting in a negative for all cases.
- Dogs were assessed before and during treatment to evaluate clinical manifestations before inclusion in the group of dogs diagnosed with non-neurological or neurological distemper.

The study also decreased the euthanasia cases related to non-neurological and neurological distemper complications after the application of AgNPs that delay the appearance of clinical signs. Euthanasia is influenced by factors related to the owner and a poor quality of life.

Another major limitation of the study is the lack of MRI results for nervous system tissue (brain and spinal cord) before and after treatment to show improvements in central nervous tissue following treatment. All these limitations highlight the need for further MRI investigations of neurological distemper cases to demonstrate the decreased neuroinflammation and safety of AgNPs. Another limitation of the study is the management of co-infections in clinical studies, as these infectious diseases can affect different systems, such as the respiratory, gastroenteric, and central nervous systems.

In the case of respiratory diseases, bacterial cultures were performed, but it is important to consider the performance of cerebrospinal fluid cultures in dogs with non-neurological and neurological distemper. In future research, bacterial cultures and molecular testing may be highlighted to demonstrate the importance of correct etiological diagnosis, as well as the most appropriate clinical management in coinfections.

## 7. Conclusions

In this clinical trial, we concluded that a treatment regimen of oral 1.5 mL/kg and nasal 0.25 mL/kg of 3% AgNPs every 8 h for a period of 15 days to dogs with clinical signs of non-neurological distemper and supportive therapy, as well as treatment for dogs with clinical signs of neurological distemper with a combination of oral 1.5 mL/kg and nasal 0.5 mL/kg of 3% AgNPs every 8 h during the first 7 days and modifications of these doses depending on the patient’s response for up to 30 days, in addition to the application of supportive therapy, had good efficacy and safety for the control of non-neurological and neurological distemper. Thus, the study indicates that AgNPs can minimize the effects of distemper and progression of complications in dogs diagnosed with non-neurological and neurological distemper, increasing quality of life in dogs that need a long-term therapeutic regimen. To our knowledge, this is the first article in the literature reporting the antiviral activity of AgNPs with significant survival in dogs diagnosed with neurological distemper. The survival rate of the experimental group with clinical signs of neurological distemper was 65.6%, significantly higher than that of the control group, which was 0%.

## Figures and Tables

**Table 1:** Prohibited medications during the study.

Medications *	Description
Interferons	Feline interferon alpha (FeIFN-α) and recombinant feline interferon omega (rFeIFN-Ω).
Immunotherapy	Autologous hyperimmune sera and heterologous hyperimmune sera.
Antivirals	Ribavirin, Boceprevir, Favipiravir and Azatioprin.
Investigational therapies	Sulfated polysaccharide, flavonoids (quercetin, morin, rutin and hesperidin), phenolic acids (cinnamic, trans-cinnamic and ferulic), mesenchymal stem cells and dialyzed leukocyte extract (DLE).
Other	Immunostimulants based on the lyophilized culture of inactivated Parapoxvirus ovis.

* The administration of these medications before study entry and during the study was considered an exclusion criterion.

**Table 2:** Schedule of the procedures for the dogs that were included in the study population according to group.

Study	Group 1a		Group 1b		Group 2a			Group 2b		
	Week		Week		Week			Week		
	0	2	0	2	0	2	4	0	2	4
Questionnaire	X	X	X	X	X	X	X	X	X	^a^
Clinical evaluation	X	X	X	X	X	X	X	X	X	^a^
Clinical chemistry	X	X	X	X	X	X	X	X	X	^a^

^a^ All the dogs in this group during this week died, or euthanasia was indicated.

**Table 3:** Blood chemistry values of dogs during treatment with AgNPs and supportive therapy.

Study	Reference	Group 1a		Group 1b		Group 2a			Group 2b		
		Week 0	Week 2	Week 0	Week 2	Week 0	Week 2	Week 4	Week 0	Week 2	Week 4
Glucose	90–150 mg/dL	107 ± 10	103 ± 10	102 ± 10	105 ± 10	101 ± 10	99 ± 10	102 ± 10	105 ± 10	101 ± 10	-----
BUN	10–58 mg/dL	23 ± 8	22 ± 8	25 ± 8	27 ± 8	24 ± 8	26 ± 8	29 ± 8	20 ± 8	25 ± 8	-----
Creatinine	0.3–1.4 mg/dL	0.9 ± 0.3	1.0 ± 0.3	1.1 ± 0.3	0.9 ± 0.3	0.8 ± 0.3	1.0 ± 0.3	1.2 ± 0.3	1.0 ± 0.3	1.2 ± 0.3	-----
Cholesterol	125–310 mg/dL	299 ± 60	290 ± 60	291 ± 60	295 ± 60	289 ± 60	292 ± 60	298 ± 60	291 ± 60	295 ± 60	-----
Triglycerides	40–150 mg/dL	65 ± 15	62 ± 15	61 ± 15	64 ± 15	60 ± 15	62 ± 15	65 ± 15	62 ± 15	64 ± 15	-----
Total protein	4.0–5.8 g/dL	4.5 ± 0.3	4.7 ± 0.3	5.1 ± 0.3	5.0 ± 0.3	4.9 ± 0.3	4.6 ± 0.3	4.7 ± 0.3	4.8 ± 0.3	5.0 ± 0.3	-----
Albumin	2.2–2.9 g/dL	2.4 ± 0.3	2.7 ± 0.3	2.5 ± 0.3	2.6 ± 0.3	2.7 ± 0.3	2.8 ± 0.3	2.6 ± 0.3	2.6 ± 0.3	2.8 ± 0.3	-----
Globulin	2.1–4.4 g/dL	2.5 ± 0.3	2.7 ± 0.3	2.4 ± 0.3	2.6 ± 0.3	2.6 ± 0.3	2.5 ± 0.3	2.7 ± 0.3	2.7 ± 0.3	2.9 ± 0.3	-----
ALP	75–450 U/L	136 ± 90	155 ± 95	140 ± 90	163 ± 100	146 ± 90	165 ± 95	189 ± 105	145 ± 90	149 ± 90	-----
ALT	10–60 U/L	30 ± 25	35 ± 28	40 ± 25	48 ± 25	39 ± 25	47 ± 30	56 ± 30	39 ± 25	43 ± 25	-----
AST	10–65 U/L	33 ± 20	43 ± 30	36 ± 20	39 ± 20	29 ± 20	38 ± 25	45 ± 30	39 ± 20	46 ± 20	-----
GGT	3–9 U/L	5 ± 2.5	7 ± 2.5	4.5 ± 2.5	6.5 ± 2.5	4.9 ± 2.5	6.1 ± 2.5	7.5 ± 3	4.8 ± 2.5	5.8 ± 2.5	-----
Total Bilirubin	0.1–1.0 mg/dL	0.5 ± 0.2	0.6 ± 0.2	0.4 ± 0.2	0.5 ± 0.2	0.5 ± 0.2	0.4 ± 0.2	0.5 ± 0.2	0.7 ± 0.2	0.6 ± 0.2	-----
Uric acid	0.2–1.6 mg/dL	0.9 ± 0.2	1.1 ± 0.2	0.6 ± 0.2	0.8 ± 0.2	0.7 ± 0.2	0.9 ± 0.2	0.8 ± 0.2	0.5 ± 0.2	0.7 ± 0.2	-----
Phosphorus	4.8–10.7 mg/dL	6.8 ± 0.6	7.1 ± 0.6	5.8 ± 0.6	6.1 ± 0.6	5.9 ± 0.6	6.1 ± 0.6	6.9 ± 0.6	7.8 ± 0.6	8.1 ± 0.6	-----
Amylase	230–500 U/L	290 ± 60	300 ± 60	310 ± 60	300 ± 60	315 ± 60	340 ± 60	320 ± 60	290 ± 60	310 ± 60	-----
Lipase	<550 U/L	380 ± 80	397 ± 80	401 ± 80	390 ± 80	389 ± 80	397 ± 80	409 ± 80	310 ± 80	370 ± 80	-----

Data presented as mean with standard deviations. BUN, blood urea nitrogen; ALP, alkaline phosphatase; ALT, alanine aminotransferase; AST, aspartate aminotransferase; GGT, gamma glutamyl transferase; ------, At week 4, all the dogs in group 2b died.

**Table 4:** Survival and death rates for the study groups.

	Group 1a	Group 1b	Group 2a	Group 2b
Total survival rate	44/52 (84.6%)	7/46 (15.2%)	38/58(65.5%)	0/51(0%)
Survival rate of dogs without sequels	39/52 (75.0%)	3/46 (6.5%)	28/58(48.3%)	0/51(0%)
Survival rate of dogs with sequels	5/52 (9.6%)	4/46 (8.7%)	10/58(17.2%)	0/51(0%)
Percentage of dogs died	8/52 (15.38%)	39/46 (84.78%)	20/58 (34.48%)	51/51 (100%)
Percentage of dogs died of the disease	7/52 (13.46%)	31/46 (67.39%)	12/58 (20.69%)	36/51 (70.59%)
Percentage of dogs died by euthanasia *	1/52 (1.92%)	8/46 (17.93%)	8/58 (13.79%)	15/51 (29.41%)

**Group 1a:** Experimental group diagnosed with non-neurological distemper treated with AgNPs and support therapy. **Group 1b:** Control group diagnosed with non-neurological distemper that only received supportive therapy. **Group 2a:** Experimental group diagnosed with neurological distemper treated with AgNPs and supportive therapy. **Group 2b:** Control group diagnosed with neurological distemper that only received supportive therapy. * Due to the general discomfort of the dog, the owner suspended treatment and decided to euthanize.

**Table 5:** Test of equality of survival distributions at different treatment levels (Groups 1a and 1b).

Overall Comparisons			
	Chi-Square	df	Sig.
Log Rank (Mantel–Cox)	48.187	1	<0.001
Breslow (Generalized Wilcoxon)	41.249	1	<0.001
Tarone–Ware	44.785	1	<0.001

The statistical results allow for the null hypothesis of no difference between groups to be rejected, with a significant difference being observed in the survival of the experimental and control groups (1a and 1b).

**Table 6:** Test of equality of survival distribution at different treatment levels (Groups 2a and 2b).

Overall Comparisons			
	Chi-Square	df	Sig.
Log Rank (Mantel–Cox)	57.062	1	<0.001

The statistical results allow for us to reject the null hypothesis of no difference between groups, with a significant difference being observed in the survival of the experimental and control groups (2a and 2b).

**Table 7:** Kaplan–Meier Method (Groups 1a and 1b).

Case Processing Summary				
			Censored	
Treatment	Total N	N of Events	N	Percent
Control	46	39	7	15.2%
AgNPs	52	8	44	84.6%
Overall	98	47	51	52.0%

The differences in means for the survival time was limited to 15 days according to treatment length.

**Table 8:** Kaplan–Meier Method (Groups 2a and 2b).

Case Processing Summary				
			Censored	
Treatment	Total N	N of Events	N	Percent
Control	58	20	38	65.5%
AgNPs	51	51	0	0.0%
Overall	109	71	38	34.9%

The difference in means for the survival time was limited to 28 days according to treatment length.

**Table 9:** Mean survival time (Groups 1a and 1b).

			Mean ^a^	
			95% Confidence Interval	
	Estimate	Std. Error	Lower Bound	Upper Bound
Control	8.783	0.597	7.613	9.952
AgNPs	13.750	0.429	12.909	14.591
Overall	11.418	0.439	10.557	12.279

^a^ Estimation was limited to the largest survival time if censored.

**Table 10:** Mean survival time (Groups 2a and 2b).

			Mean ^a^	
			95% Confidence Interval	
	Estimate	Std. Error	Lower Bound	Upper Bound
Control	22.500	1.121	2.304	24.696
AgNPs	11.941	0.966	10.047	13.835
Overall	17.560	0.900	15.795	19.324

^a^ Estimation is limited to the largest survival time if censored.

## Data Availability

The datasheets supporting the conclusions of this article are included within the article.

## Supplementary Materials

The following supporting information can be downloaded at: https://www.mdpi.com/article/10.3390/v14112329/s1, Tables S1: Support therapy for the treatment of dogs diagnosed with non-neurological and neurological distemper; Tables S2: Characteristics and results obtained for the dogs with non-neurological distemper treated with AgNPs (Group 1a); Tables S3: Characteristics and results obtained for the dogs with non-neurological distemper treated with only supportive therapy (Group 1b); Table S4: Characteristics and results obtained for the dogs with neurological distemper treated with AgNPs (Group 2a); Table S5: Characteristics and results obtained for the dogs with neurological distemper treated with only supportive therapy (Group 2b).

## References

[1] Willi B., Spiri A.M., Meli M.L., Grimm F., Beatrice L., Riond B., Bley T., Jordi R., Dennler M., Hofmann-Lehmann R. (2015). Clinical and Molecular Investigation of a Canine Distemper Outbreak and Vector-Borne Infections in a Group of Rescue Dogs Imported from Hungary to Switzerland. BMC Vet. Res..

[2] Lednicky J.A., Dubach J., Kinsel M.J., Meehan T.P., Bocchetta M., Hungerford L.L., Sarich N.A., Witecki K.E., Braid M.D., Pedrak C. (2004). Genetically Distant American Canine Distemper Virus Lineages Have Recently Caused Epizootics with Somewhat Different Characteristics in Raccoons Living around a Large Suburban Zoo in the USA. Virol. J..

[3] Beineke A., Puff C., Seehusen F., Baumgärtner W. (2009). Pathogenesis and Immunopathology of Systemic and Nervous Canine Distemper. Vet. Immunol. Immunopathol..

[4] Rendon-Marin S., Da Fontoura Budaszewski R., Canal C.W., Ruiz-Saenz J. (2019). Tropism and Molecular Pathogenesis of Canine Distemper Virus. Virol. J..

[5] Deem S.L., Spelman L.H., Yates R.A., Montali R.J. (2000). Canine Distemper in Terrestrial Carnivores: A Review. J. Zoo Wildl. Med..

[6] Koutinas A.F., Baumgärtner W., Tontis D., Polizopoulou Z., Saridomichelakis M.N., Lekkas S. (2004). Histopathology and Immunohistochemistry of Canine Distemper Virus-Induced Footpad Hyperkeratosis (Hard Pad Disease) in Dogs with Natural Canine Distemper. Vet. Pathol..

[7] Beineke A., Baumgärtner W., Wohlsein P. (2015). Cross-Species Transmission of Canine Distemper Virus-an Update. One Health.

[8] Liu P.C., Chen C.A., Chen C.M., Yen C.H., Lee M.H., Chuang C.K., Tu C.F., Su B.L. (2016). Application of Xenogeneic Anti-Canine Distemper Virus Antibodies in Treatment of Canine Distemper Puppies. J. Small Anim. Pract..

[9] Martella V., Elia G., Buonavoglia C. (2008). Canine Distemper Virus. Vet. Clin. N. Am. Small Anim. Pract..

[10] Gonçalves D.S.V., Gomes M.V.S., Guterra V.L.P., Lucchi-Rodrigues A.F., Mathias C.H.T., Maestri L.F.P., Argôlo-Neto N.M., Monteiro B.S. (2018). Mesenchymal Stem Cell Infusion for the Treatment of Neurological Sequelae of Canine Distemper Virus: A Clinical Study. Genet. Mol. Res..

[11] Loots A.K., Mitchell E., Dalton D.L., Kotzé A., Venter E.H. (2017). Advances in Canine Distemper Virus Pathogenesis Research: A Wildlife Perspective. J. Gen. Virol..

[12] Tipold A., Vandevelde M., Jaggy A. (1992). Neurological Manifestations of Canine Distemper Virus Infection. J. Small Anim. Pract..

[13] Rubin S., Carr A. (2006). Canine Internal Medicine Secrets E-Book.

[14] Cunha R.D.S., da Silva Junior C.L., Costa C.A., de Aguiar H.M., Junqueira Júnior D.G. (2020). Comparison of Immunity against Canine Distemper, Adenovirus and Parvovirus after Vaccination with Two Multivalent Canine Vaccines. Vet. Med. Sci..

[15] Zacarias J., Dimande A., Achá S., Dias P.T., Leonel E.M., Messa A., Macucule B., Júnior J.L., Bila C.G. (2016). Severe Canine Distemper Outbreak in Unvaccinated Dogs in Mozambique. J. S. Afr. Vet. Assoc..

[16] Garde E., Pérez G., Acosta-Jamett G., Bronsvoort B.M. (2013). Characteristics of a Canine Distemper Virus Outbreak in Dichato, Chile Following the February 2010 Earthquake. Animals.

[17] Elia G., Belloli C., Cirone F., Lucente M.S., Caruso M., Martella V., Decaro N., Buonavoglia C., Ormas P. (2008). In Vitro Efficacy of Ribavirin against Canine Distemper Virus. Antivir. Res..

[18] Lanave G., Cavalli A., Martella V., Fontana T., Losappio R., Tempesta M., Decaro N., Buonavoglia D., Camero M. (2017). Ribavirin and Boceprevir Are Able to Reduce Canine Distemper Virus Growth in Vitro. J. Virol. Methods.

[19] Carvalho O.V., Saraiva G.L., Ferreira C.G.T., Felix D.M., Fietto J.L.R., Bressan G.C., Almeida M.R., Silva Júnior A. (2014). In-Vitro Antiviral Efficacy of Ribavirin and Interferon-Alpha against Canine Distemper Virus. Can. J. Vet. Res..

[20] Wang H., Jia X., Yang L., Sun L., Wang H., Liu W. (2008). [Comparison of antiviral activity between FeIFN-omega and FeIFN-alpha]. Sheng Wu Gong Cheng Xue Bao.

[21] Xue X., Zhu Y., Yan L., Wong G., Sun P., Zheng X., Xia X. (2019). Antiviral Efficacy of Favipiravir against Canine Distemper Virus Infection in Vitro. BMC Vet. Res..

[22] Carvalho O.V., Botelho C.V., Ferreira C.G.T., Ferreira H.C.C., Santos M.R., Diaz M.A.N., Oliveira T.T., Soares-Martins J.A.P., Almeida M.R., Silva Júnior A. (2013). In Vitro Inhibition of Canine Distemper Virus by Flavonoids and Phenolic Acids: Implications of Structural Differences for Antiviral Design. Res. Vet. Sci..

[23] Zhang J., Cui D., Zuo Y., Zheng Z., Wu F., Li W., Zhang Y., Huo S., Li N., Li L. (2021). Donkey-Derived Anti-CDV IgG, as a Passive Immunotherapy Agent, Can Effectively Increase Survival Rates of the Experimental CDV-Infected Dogs. BMC Vet. Res..

[24] Bogdanchikova N., Vázquez-Muñoz R., Huerta-saquero A., Pena-Jasso A., Aguilar-Uzcanga G., Picos-díaz P.L., Pestryakov A., Burmistrov V., Martynyuk O., Luna-Vázquez-Gómez R. (2016). Silver Nanoparticles Composition for Treatment of Distemper in Dogs. Int. J. Nanotechnol..

[25] Youssef F.S., El-Banna H.A., Elzorba H.Y., Galal A.M. (2019). Application of Some Nanoparticles in the Field of Veterinary Medicine. Int. J. Vet. Sci. Med..

[26] Percie du Sert N., Hurst V., Ahluwalia A., Alam S., Avey M.T., Baker M., Browne W.J., Clark A., Cuthill I.C., Dirnagl U. (2020). The ARRIVE Guidelines 2.0: Updated Guidelines for Reporting Animal Research*. J. Cereb. Blood Flow Metab..

[27] Pinheiro A.O., Cardoso M.T., Vidane A.S., Casals J.B., Passarelli D., Alencar A.L.F., Sousa R.L.M., Fantinato-Neto P., Oliveira V.C., Lara V.M. (2016). Controversial Results of Therapy with Mesenchymal Stem Cells in the Acute Phase of Canine Distemper Disease. Genet. Mol. Res..

[28] Sedgwick P. (2012). Why Randomise in Clinical Trials?. BMJ.

[29] Schulz K.F., Altman D.G., Moher D. (2010). CONSORT 2010 Statement: Updated Guidelines for Reporting Parallel Group Randomised Trials. J. Pharmacol. Pharmacother..

[30] Kendall M.J. (2003). Designing a Research Project: Randomised Controlled Trials and Their Principles. Emerg. Med. J..

[31] Ranjan R., Kumar Jha A., Kumar S. (2021). Canine Distemper: A Fatal Disease Seeking Special Intervention. J. Entomol. Zool. Stud..

[32] Sykes J. (2022). Greene’s Infectious Diseases of the Dog and Cat.

[33] Nelson R., Couto C. (2019). Small Animal Internal Medicine-E-Book.

[34] Galán A., Gamito A., Carletti B.E., Guisado A., De Las Mulas J.M., Pérez J., Martín E.M. (2014). Case Report Rapport de Cas Uncommon Acute Neurologic Presentation of Canine Distemper in 4 Adult Dogs. Can. Vet. J..

[35] Couto R.M., França S.A., Rios M.A., Rosado I.R., Costa P.M., Ecco R. (2013). Clinical and Pathological Findings of Necrotizing Meningoencephalitis in a Maltese Dog. Braz. J. Vet. Pathol..

[36] Candini D., Biasato I., Dell’Armelina Rocha P.R., Grego E., Capucchio M.T., Vercelli C. (2017). How Behavioral Changes Can Indicate Serious Cerebral Pathology: A Case Report of Concomitant Olfactory Neuroblastoma and Distemper Virus Encephalitis in a Swiss Shepherd Dog. Vet. Sci..

[37] Ranjithkumar M. (2021). Clinical Progression of Naturally Occurring Canine Distemper Encephalomyelitis Cases. Indian J. Vet. Med..

[38] Richards T.R., Whelan N.C., Pinard C.L., Alcala F.C., Wolfe K.C. (2011). Optic Neuritis Caused by Canine Distemper Virus in a Jack Russell Terrier. Can. Vet. J..

[39] di Francesco C.E., di Francesco D., di Martino B., Speranza R., Santori D., Boari A., Marsilio F. (2012). Detection by Hemi-Nested Reverse Transcription Polymerase Chain Reaction and Genetic Characterization of Wild Type Strains of Canine Distemper Virus in Suspected Infected Dogs. J. Vet. Diagn. Investig..

[40] Zhang H., Shan F., Zhou X., Li B., Zhai J.Q., Zou S.Z., Wu M.F., Chen W., Zhai S.L., Luo M.L. (2017). Outbreak and Genotyping of Canine Distemper Virus in Captive Siberian Tigers and Red Pandas. Sci. Rep..

[41] Sun L., Singh A., Vig K., Pillai S.R., Singh S.R. (2008). Silver Nanoparticles Inhibit Replication of Respiratory Syncytial Virus. J. Biomed. Nanotechnol..

[42] Morris D., Ansar M., Speshock J., Ivanciuc T., Qu Y., Casola A., Garofalo R. (2019). Antiviral and Immunomodulatory Activity of Silver Nanoparticles in Experimental Rsv Infection. Viruses.

[43] Gaikwad S., Ingle A., Gade A., Rai M., Falanga A., Incoronato N., Russo L., Galdiero S., Galdiero M. (2013). Antiviral Activity of Mycosynthesized Silver Nanoparticles against Herpes Simplex Virus and Human Parainfluenza Virus Type 3. Int. J. Nanomed..

[44] Yin J.J., Li X.J., Zheng C.L. (2013). Potential Mechanism and Inhibitory Effects of Silver Nanoparticles on Parainfluenza Virus Type 3. J. Jiangsu Univ..

[45] Khandelwal N., Kaur G., Chaubey K.K., Singh P., Sharma S., Tiwari A., Singh S.V., Kumar N. (2014). Silver Nanoparticles Impair Peste Des Petits Ruminants Virus Replication. Virus Res..

[46] Summers B.A., Greisen H.A., Appel M.J. (1984). Canine Distemper Encephalomyelitis: Variation with Virus Strain. J. Comp. Pathol..

[47] Krakowka S., Olsen R., Confer A., Koestner A., Mccullough B. (1975). Serologic Response to Canine Distemper Viral Antigens in Gnotobiotic Dogs Infected with Canine Distemper Virus. J. Infect. Dis..

[48] Vandevelde M., Zurbriggen A. (1995). The Neurobiology of Canine Distemper Virus Infection. Vet. Microbiol..

[49] Blixenkrone-Moller M., Pedersen I.R., Appel M.J., Griot C. (1991). Detection of IgM Antibodies against Canine Distemper Virus in Dog and Mink Sera Employing Enzyme-Linked Immunosorbent Assay (ELISA). J. Vet. Diagn. Investig..

[50] von Messling V., Harder T.C., Moennig V., Rautenberg P., Nolte I., Haas L. (1999). Rapid and Sensitive Detection of Immunoglobulin M (IgM) and IgG Antibodies against Canine Distemper Virus by a New Recombinant Nucleocapsid Protein-Based Enzyme-Linked Immunosorbent Assay. J. Clin. Microbiol..

[51] Jóźwik A., Frymus T., Mizak B., Rzezutka A. (2004). Antibody Titres against Canine Distemper Virus in Vaccinated and Unvaccinated Dogs. J. Vet. Med. Ser. B Infect. Dis. Vet. Public Health.

[52] Borrego B., Lorenzo G., Mota-Morales J.D., Almanza-Reyes H., Mateos F., López-Gil E., de la Losa N., Burmistrov V.A., Pestryakov A.N., Brun A. (2016). Potential Application of Silver Nanoparticles to Control the Infectivity of Rift Valley Fever Virus in Vitro and in Vivo. Nanomedicine.

[53] Nefedova E., Koptev V., Bobikova A.S., Cherepushkina V., Mironova T., Afonyushkin V., Shkil N., Donchenko N., Kozlova Y., Sigareva N. (2021). The Infectious Bronchitis Coronavirus Pneumonia Model Presenting a Novel Insight for the Sars-Cov-2 Dissemination Route. Vet. Sci..

[54] Almanza-Reyes H., Moreno S., Plascencia-López I., Alvarado-Vera M., Patrón-Romero L., Borrego B., Reyes-Escamilla A., Valencia-Manzo D., Brun A., Pestryakov A. (2021). Evaluation of Silver Nanoparticles for the Prevention of SARS-CoV-2 Infection in Health Workers: In Vitro and in Vivo. PLoS ONE.

[55] Fighera R.A., Souza T.M., Silva M.C., Brum J.S., Graça D.L., Kommers G.D., Irigoyen L.F., Barros C.S.L. (2008). Causas de Morte e Razões Para Eutanásia de Cães Da Mesorregião Do Centro Ocidental Rio-Grandense. Pesqui. Veterinária Bras..

[56] Koutinas A.F., Polizopoulou Z.S., Baumgaertner W., Lekkas S., Kontos V. (2002). Relation of Clinical Signs to Pathological Changes in 19 Cases of Canine Distemper Encephalomyelitis. J. Comp. Pathol..

[57] Silva M.C., Fighera R.A., Mazzanti A., Brum J.S., Pierezan F., Barros C.S.L. (2009). Neuropatologia Da Cinomose Canina: 70 Casos (2005–2008)^1^. Pesq. Vet. Bras..

[58] Krakowka S., Koestner A. (1976). Age-Related Susceptibility to Infection with Canine Distemper Virus in Gnotobiotic Dogs. J. Infect. Dis..

[59] Study A.I., Vandevelde M., Fankhauser R., Kristensen F., Kristensen B. (1981). Acta Neuropathologica Immunoglobulins in Demyelinating Lesions in Canine Distemper Encephalitis. Acta Neuropathol..

[60] Giulia D., Francesca V., Sandra S., Alessandra S., Gualtiero G., Luciana G., Laura C. (2006). A Molecular Study of Hippocampus in Dogs with Convulsion during Canine Distemper Virus Encephalitis. Brain Res..

[61] Vandevelde M., Zurbriggen A., Higgins R.J., Palmer D. (1985). Spread and Distribution of Viral Antigen in Nervous Canine Distemper*.

[62] Clausen M.B., Bandholm T., Rathleff M.S., Christensen K.B., Zebis M.K., Graven-Nielsen T., Hölmich P., Thorborg K. (2018). The Strengthening Exercises in Shoulder Impingement Trial (The SExSI-Trial) Investigating the Effectiveness of a Simple Add-on Shoulder Strengthening Exercise Programme in Patients with Long-Lasting Subacromial Impingement Syndrome: Study Protocol for a Pragmatic, Assessor Blinded, Parallel-Group, Randomised, Controlled Trial. Trials.

[63] Andersen A.L., Houlind M.B., Nielsen R.L., Jørgensen L.M., Treldal C., Damgaard M., Bengaard A.K., Juul-Larsen H.G., Laursen L.B., Iversen E. (2021). Optimization of Nutrition And Medication (OptiNAM) for Acutely Admitted Older Patients: Protocol for a Randomized Single-Blinded Controlled Trial. Trials.

[64] Bhandari M., Busse J.W., Jackowski D., Montori V.M., Schünemann H., Sprague S., Mears D., Schemitsch E.H., Heels-Ansdell D., Devereaux P.J. (2004). Association between Industry Funding and Statistically Significant Pro-Industry Findings in Medical and Surgical Randomized Trials. Can. Med. Assoc. J..

[65] Anderson M.L., Chiswell K., Peterson E.D., Tasneem A., Topping J., Califf R.M. (2015). Compliance with Results Reporting at ClinicalTrials.Gov. N. Engl. J. Med..

[66] Lexchin J., Bero L.A., Djulbegovic B., Clark O. (2003). Pharmaceutical Industry Sponsorship and Research Outcome and Quality: Systematic Review. BMJ.

[67] Lundh A., Lexchin J., Mintzes B., Schroll J.B., Bero L. (2017). Industry Sponsorship and Research Outcome. Cochrane Database Syst. Rev..

[68] Okike K., Kocher M.S., Wei E.X., Mehlman C.T., Bhandari M. (2009). Accuracy of Conflict-of-Interest Disclosures Reported by Physicians. N. Engl. J. Med..

[69] Dickersin K., Chalmers I. (2011). Recognizing, Investigating and Dealingwith Incomplete and Biased Reporting of Clinical Research: From Francis Bacon to the WHO. J. R. Soc. Med..

[70] Schott G., Pachl H., Limbach U., Gundert-Remy U., Ludwig W.D., Lieb K. (2010). Finanzierung von Arzneimittelstudien Durch Pharmazeutische Unternehmen Und Die Folgen. Teil.

[71] Harmon C.P., Chalmers P.N., Carpiniello M.F., Cvetanovich G.L., Cole B.J., Bach B.R. (2016). Inconsistencies between Physician-Reported Disclosures at the AAOS Annual Meeting and Industry-Reported Financial Disclosures in the Open Payments Database. J. Bone Jt. Surg. Am..

[72] Lane P. (2008). Handling Drop-out in Longitudinal Clinical Trials: A Comparison of the LOCF and MMRM Approaches. Pharm. Stat. J. Appl. Stat..

