# Supplementary material for: Evaluation of the Efficacy and Safety of Silver Nanoparticles in the Treatment of Non-Neurological and Neurological Distemper in Dogs: A Randomized Clinical Trial
Source: Viruses. 2022 Oct 24;14(11):2329. doi: 10.3390/v14112329 (PMC9694365; doi:10.3390/v14112329)
Supplement: Supplementary file 1 [file viruses-14-02329-s001.zip › Table S1.pdf]

Table S1. Support therapy for the treatment of dogs diagnosed with non-neurological and neurological distemper.

Drug	Dose (mg/Kg)	Route of administration	Interval between doses (hours)	Treatment duration (days)
**Antimicrobials**				
Ampicillin-amoxicillin	20	Oral, IV, SC	8	7
Doxycycline	5-10	Oral, IV	12	7
Chloramphenicol	15-25	Oral, SC	8	7
Florfenicol	25-50	SC, IM	8	3-5
Cephapirin	10-30	IM, IV, SC	6-8	3-5
Amoxicillin–clavulanic acid	12.5-25	Oral	12	7
**Antipyretics**				
Meloxicam[a]	0.2[d], 0.1[c]	Oral, IV, SC	24	4-5
**Antiemetics**				
Maropitant	1	SC, IV	24	5
**Anticonvulsants**				
Phenobarbital	2	Oral, IV, IM	12	As needed
Pregabalin	3-4	Oral	8	As needed
Levetiracetam	20	Oral	6	As needed
Diazepam	0.2	IV	8	As needed
**Anti-inflammatories**				
Dexamethasone				
CNS edema	1-2	IV	24	1
Optic neuritis	0.1	Oral, IV, SC	24	3-5
Prednisone	2	Oral	24	7
**Vitamins**				
Vitamin A	10,000[d]	Oral	24	7
Vitamin E	100-400[e]	Oral	12	7
Vitamin B12	0.025-0.25[f]	V, IM, SC	24	7

[a]: They are justified with temperatures above 40°C; [b]: Only the first day; [c]: Continue through days 2 to 5. [d]: UI (International Unit); [e]:mg(milligrams); [f]:pg(picograms); SC: subcutaneous; IV: intravenous; IM: intramuscular. Source: modified from [32,33,35].
