# Supplementary material for: Evaluation of the Efficacy and Safety of Silver Nanoparticles in the Treatment of Non-Neurological and Neurological Distemper in Dogs: A Randomized Clinical Trial
Source: Viruses. 2022 Oct 24;14(11):2329. doi: 10.3390/v14112329 (PMC9694365; doi:10.3390/v14112329)
Supplement: Supplementary file 1 [file viruses-14-02329-s001.zip › Table S2.pdf]

Table S2. Characteristics and results obtained for the dogs with non-neurological distemper treated with AgNPs (Group 1a).

No.	Breed	Sex	Age (months)	Weight (kg)	IgM Value	Temp. (ºC)	RT-PCR	Clinical signs	Report
1	Boston Terrier	M	36	14	1:160	40.1	Positive	Respiratory disease, chronic cough, tracheobronchitis, eye discharge and did not respond to antibiotics.	Recovered without sequelae.
2	American Pit Bull	M	3	3.2	1:80	40.0	Positive	Respiratory, runny nose, conjunctivitis and keratosis.	Recovered without sequelae.
3	Creole	F	4	3	1:160	39.9	Positive	Respiratory, neurological, conjunctivitis, melena.	Recovered without sequelae.
4	Chihuahua	M	5	2.2	1:320	39.8	Positive	Respiratory, cachexia, corneal opacity, conjunctivitis and keratosis.	Recovered without sequelae.
5	Chihuahua Mix.	F	8	4.5	1:40	39.9	Positive	Respiratory, conjunctivitis, keratosis and cachexia.	Died.
6	German Shepherd	F	6	20	1:160	40.0	Positive	Respiratory, runny nose, conjunctivitis, keratosis and cachexia.	Recovered without sequelae.
7	Mixed	M	8	18	1:160	40.1	Positive	Respiratory, digestive, conjunctivitis, melena, keratoconjunctivitis, jaw tics.	Recovered with neurologi

									cal sequelae.
8	Poodle Mix.	M	60	11	1:320	40.3	Positive	Respiratory, cachexia, conjunctivitis, keratosis, and corneal opacity.	Died.
9	Creole	F	6	6	1:80	39.6	Positive	Respiratory, cachexia, conjunctivitis, cough, keratosis.	Recovered without sequelae.
10	Sharpei	F	60	22	1:80	39.9	Positive	Respiratory, keratosis and conjunctivitis and runny nose.	Recovered without sequelae.
11	Schnauzer	M	6	5	1:160	39.5	Positive	Bacterial respiratory disease, cough and runny nose.	Recovered without sequelae.
12	Weimaraner	M	84	35	n/d	39.5º	Positive	Respiratory disease, persistent cough, and laryngotracheitis.	Recovered without sequelae.
13	Creole	F	15	12	1:160	40.5	Positive	Respiratory, digestive, diarrhea, conjunctivitis and keratosis	Recovered without sequelae.
14	Dachshund	F	30	4.5	1:80	39.6	Positive	Respiratory, nasal and ocular discharge and keratosis.	Died.
15	Scottish Terrier	M	120	9	1:320	40.3	Positive	Digestive, cachexia, bloody diarrhea and abdominal spasm.	Recovered without sequelae.

16	Pit Bull	F	24	24	1:160	41.0	Positive	Digestive, diarrhea, vomiting, keratoconjunctivitis, corneal opacity, myoclonus.	Recovered with neurological sequelae.
17	Chihuahua Mix	M	132	2.8	1:160	39.9	Positive	Respiratory, digestive, conjunctivitis and corneal opacity, keratosis and melena.	Recovered without sequelae.
18	Maltese	M	48	8	1:160	40.8	Positive	Digestive, melena diarrhea and keratosis, cachexia	Recovered without sequelae.
19	Doberman	F	72	34	1:320	40.5	Positive	Respiratory, keratoconjunctivitis, throat inflammation and cough.	Recovered without sequelae.
20	Poodle	F	5	3	1:40	40.3	Positive	Respiratory, keratoconjunctivitis,	Recovered without sequelae.
21	Chihuahua	F	12	2.5	1:40	40.4	Positive	Respiratory, digestive, diarrhea with melena and prostration.	Died.
22	Creole	M	22	26	1:80	37.1	Positive	Respiratory and digestive disease, cachexic, runny nose and mucous diarrhea.	Died.
23	German Shepherd	F	12	28	1:160	40.1	Positive	Respiratory, anorexia, cachexia, keratoconjunctivitis and airway inflammation.	Recovered without sequelae.
24	Chihuahua	M	60	3.5	1:320	39.9	Positive	Respiratory, runny nose, ophthalmic, cough, keratosis and melena.	Recovered

									without sequelae.
25	Weimaraner	F	48	32	1:160	40.4	Positive	Chronic respiratory disease, cough, runny nose, did not respond to antibiotics.	Recovered without sequelae.
26	Mixed	F	84	6	1:160	39.6	Positive	Dorsal abscess, septicemia, anorexia, did not respond to antibiotics.	Recovered without sequelae.
27	P. Australian	M	96	16	1:80	39.6	Positive	Respiratory disease, throat inflammation and trachea bronchitis.	Recovered without sequelae.
28	Golden Retriever	M	48	28	1:160	40.0	Positive	Respiratory, anorexia, conjunctivitis, melena, ataxia and convulsive.	No recovered, Euthanasia*.
29	Dalmatian	M	36	24	1:80	40.2	Positive	Respiratory, conjunctivitis, cough, runny nose and conjunctivitis.	Recovered without sequelae.
30	Cocker	M	84	12	1:160	39.7	Positive	Respiratory, digestive, diarrhea with melena, and prostration.	Recovered without sequelae.
31	Pit Bull	F	24	22	1:320	39.9	Positive	Colibacillosis bacterial enteritis, bloody diarrhea / dehydration mucosa.	Recovered without sequelae.

32	English Bulldog	M	24	18	1:80	39.6	Positive	Bacterial respiratory disease, runny nose, and cough.	Recovered without sequelae.
33	Chow Chow	F	84	23	1:160	39.8	Positive	Respiratory, prostration, nasal discharge and cough.	Recovered without sequelae.
34	Chihuahua	M	6	2.8	1:80	40.3	Positive	Respiratory, corneal opacity, conjunctivitis, keratosis and melena.	Died.
35	German Shorthaired Pointer	M	48	25	1:40	40.5	Positive	Respiratory, digestive, nasal and ophthalmic discharge, melena and abdominal pain.	Recovered without sequelae.
36	Labrador	F	120	26	1:160	40.4	Positive	Respiratory, swollen glands and throat, keratosis, conjunctivitis and cachexia.	Recovered without sequelae.
37	Pit Bull	M	15	28	1:80	39.8	Positive	Respiratory, anorexia, ataxia, nasal and ophthalmic discharge.	Recovered without sequelae.
38	Mixed	M	48	19	1:160	41.1	Positive	Respiratory, digestive, diarrhea, melena, cachexia and conjunctivitis.	Recovered without sequelae.
39	Creole	F	108	12	1:160	39.7	Positive	Respiratory, anorexia, cachexia, general pain and keratoconjunctivitis.	Recovered without sequelae.

40	Scottish	F	96	9	1:80	40.3	Positive	Respiratory, digestive, cachexia, keratoconjunctivitis melena.	Recovered without sequelae.
41	Creole	M	24	5	1:160	40.3	Positive	Respiratory, cough, runny nose, keratosis, myoclonus.	Recovered with neurological sequelae.
42	Schnauzer	M	48	5.6	1:160	40.4	Positive	Respiratory, runny nose, corneal opacity and keratoconjunctivitis.	Recovered without sequelae.
43	Mixed	F	21	30	1:80	39.5	Positive	Respiratory, conjunctivitis, corneal opacity, keratosis.	Recovered without sequelae.
44	German Shepherd	M	16	33	1:80	40.0	Positive	Respiratory, runny nose, keratoconjunctivitis, plantar keratosis, cachexia, and myoclonus.	Recovered with neurological sequelae.
45	Maltese	F	12	7	1:320	40.1	Positive	Respiratory, conjunctivitis, melena and keratosis.	Recovered without sequelae.
46	Mixed	M	84	18	1:160	40.5	Positive	Respiratory, digestive, conjunctivitis, diarrhea, with melena and vomiting.	Recovered without sequelae.

47	Poodle	M	18	6	1:320	39.7	Positive	Respiratory, keratoconjunctivitis, eye discharge and nasal melena.	Recovered without sequelae.
48	Pit Bull	F	32	24	1:160	41.0	Positive	Digestive, cachexia, conjunctivitis, diarrhea, corneal opacity, jaw tics.	Recovered with neurological sequelae.
49	Belgian Shepherd	F	48	22	1:160	39.7	Positive	Respiratory, cachexia, keratoconjunctivitis and throat inflammation.	Recovered without sequelae.
50	Rottweiler	M	36	31	1:80	40.2	Positive	Digestive, cachexia, diarrhea, melena and keratoconjunctivitis.	Died.
51	Chihuahua	M	60	4	1:80	39.8	Positive	Respiratory, runny nose, tracheobronchitis, conjunctivitis, cachexia,	Recovered without sequelae.
52	Mixed	M	24	11	1:80	40.2	Positive	Respiratory, digestive, cachexia, conjunctivitis, melena and prostration.	Recovered without sequelae.

M: Male, F: Female, Kg: Kilogram, IgM: Virus-specific immunoglobulin M/Value of immunoglobulin M, Indirect Immunofluorescent Assay (IFA) test was used for the quantification of the IgM, Temp.: Temperature, RT-PCR: Reverse Transcriptase Polymerase Chain Reaction, *Due to the general discomfort of the dog, the owner suspended the treatment and decided to euthanize.
