# Supplementary material for: Evaluation of the Efficacy and Safety of Silver Nanoparticles in the Treatment of Non-Neurological and Neurological Distemper in Dogs: A Randomized Clinical Trial
Source: Viruses. 2022 Oct 24;14(11):2329. doi: 10.3390/v14112329 (PMC9694365; doi:10.3390/v14112329)
Supplement: Supplementary file 1 [file viruses-14-02329-s001.zip › Table S3.pdf]

Table S3. Characteristics and results obtained for the dogs with non-neurological distemper treated with only supportive therapy (Group 1b).

No.	Breed	Sex	Age (months)	Weight (kg)	IgM Value	Temp. (ºC)	RT-PCR	Clinical signs	Report
1	Scottish Terrier	M	112	11	1:40	39.8	Positive	Respiratory, keratoconjunctivitis, runny nose, cachexia, posterior myoclonus in thoracic and maxillofacial limbs.	Died.
2	Golden Retriever cross	F	120	19	1:160	39.6	Positive	Respiratory, weight loss, conjunctivitis, melena, evolved to seizures.	Recovered without sequelae.
3	Pit Bull	F	6	16	1:80	39.7	Positive	Digestive, headaches, cachexia, keratoconjunctivitis, melena, corneal ulcer, later convulsive.	Died.
4	Golden Retriever	M	18	22	1:160	40.2	Positive	Respiratory, conjunctivitis, corneal opacity, cachexia, nasal and plantar keratosis, subsequent complete prostration.	Died.
5	German Shepherd	M	48	36	1:80	39.9	Positive	Respiratory, conjunctivitis, ataxia, later convulsive.	Died.
6	Cross German Shepherd and Belgian Shepherd	F	24	23	1:40	40.0	Positive	Respiratory, keratoconjunctivitis, cachexia, plantar keratosis, evolved to seizures.	Died.
7	Creole	M	15	18	1:360	39.5	Positive	Digestive, melena, weight loss, lethargy, subsequent prostration and diaphragm myoclonus.	Recovered without sequelae.

8	Creole	F	48	10	1:80	39.8	Positive	Respiratory, corneal ulcer, secretions within the nasolacrimal duct, cachexia, nasal keratosis, posterior ataxia and prostration.	Euthanasia*.
9	Poodle	F	84	6.4	1:80	40.5	Positive	Respiratory, cachexia, decayed, melena, runny nose and nasal and plantar keratosis.	Died.
10	Chihuahua cross	M	36	4.9	1:40	40.0	Positive	Respiratory, melena, corneal opacity, headache, plantar keratosis, evolved to seizures.	Died.
11	American Pit Bull	M	60	26	1:160	38.8	Positive	Respiratory, lethargy, cachexia, conjunctivitis with discharge, melena, posterior myoclonus in the pelvic limbs.	Died.
12	Pit Bull mix	F	24	18	1:80	41.0	Positive	Respiratory, corneal opacity, nasal and plantar keratosis, posterior general myoclonus.	Euthanasia.
13	Creole	M	12	12	1:40	39.5	Positive	Respiratory, nasal discharge and keratosis, corneal opacity, evolved to seizures.	Died.
14	Creole	M	72	30	1:160	39.9	Positive	Digestive, melena, cachexia, keratoconjunctivitis, posterior mandibular tics and loss of swallowing reflex.	Died.
15	Mix Maltese Poodle	F	60	8	1:160	40.2	Positive	Respiratory, weight loss, melena, evolved to seizures.	Euthanasia*.

16	Cross Schnauzer	F	21	7	1:320	39.5	Positive	Digestive, melena, cachexia, decay, keratosis, evolved to seizures.	Recovered without sequelae.
17	Cross Pug - Chihuahua	M	24	5.5	1:80	39.9	Positive	Respiratory, nasal and ophthalmic discharge, cachexia, evolved to seizures.	Died.
18	Boston Terrier	F	84	14	1:40	40.0	Positive	Respiratory, conjunctivitis, nasal and plantar keratosis, evolved to seizures.	Died.
19	Belgian Shepherd	F	36	21	1:160	40.0	Positive	Respiratory, weight loss, conjunctivitis with discharge, keratosis, later parietal and jaw tics.	Recovered with neurological sequelae.
20	Creole	M	n/d	8.5	1:160	39.7	Positive	Respiratory and digestive, melena, runny nose, keratosis, cachexia, posterior myoclonus and prostration.	Euthanasia*.
21	Creole.	M	42	23	1:320	40.3	Positive	Digestive, melena, weight loss, keratosis, evolved to myoclonus.	Euthanasia*.
22	Poodle mix	M	72	7	1:40	39.8	Positive	Respiratory, cachexia, runny nose and keratoconjunctivitis, evolved to myoclonus.	Died.
23	Husky cross	F	18	16	1:80	40.5	Positive	Respiratory, weight loss, nasal discharge, plantar keratosis, evolved to seizures.	Died.
24	Creole	F	36	24	1:320	40.1	Positive	Respiratory and digestive, melena and keratoconjunctivi	Died.

								tis, evolved to general neurological.	
25	Belgian Shepherd	F	72	21	1:160	39.6	Positive	Digestive, cachexia, melena, later prostration.	Died.
26	Pug	M	120	6.3	1:80	39.4	Positive	Respiratory, keratoconjunctivitis, cachexia, posterior prostration.	Euthanasia*.
27	Creole	F	108	19	1:40	40.1	Positive	Digestive, weight loss, corneal opacity, melena, prostration.	Died.
28	Pit Bull mix	M	24	26	1:160	39.9	Positive	Respiratory and digestive, melena, cachexia, corneal opacity, evolved to myoclonus.	Died.
29	Cross Chihuahua.	F	60	5.3	1:160	39.8	Positive	Respiratory, corneal opacity, discharge and nasal keratosis, evolved to seizures.	Died.
30	Weimaraner	F	84	32	1:40	40.0	Positive	Digestive, nasal keratosis, melena, keratoconjunctivitis, evolved to neurological with myoclonus.	Died.
31	Creole	M	48	16	1:80	40.2	Positive	Respiratory, corneal opacity, nasal and ophthalmic discharge, plantar keratosis, weight loss, progressed to myoclonus and seizures.	Died.
32	Creole	M	36	22	1:160	39.8	Positive	Respiratory, digestive, with weight loss, melena, keratoconjunctivitis, evolved to seizures.	Died.
33	Cross German Shepherd.	F	72	27	1:320	40.3	Positive	Respiratory, weight loss, nasal and ophthalmic	Recovered with neurologic

								discharge, nasal and plantar keratosis, evolved to myoclonus and prostration.	al sequelae.
34	Creole	F	96	24	1:160	40.0	Positive	Respiratory, digestive, cachexia, keratosis, melena, evolved to ataxia.	Recovered with neurological sequelae.
35	Husky	M	120	24	1:80	39.9	Positive	Digestive, cachexia, melena, keratosis, evolved to myoclonus and prostration.	Euthanasia*.
36	Maltese	M	108	6	1:80	39.7	Positive	Respiratory, runny nose, keratoconjunctivitis, evolved to seizures.	Died.
37	Belgian Shepherd cross	M	12	18	1:160	40.2	Positive	Respiratory, digestive, melena, conjunctivitis, nasal and ophthalmic discharge, cachexia, evolved to myoclonus.	Euthanasia*.
38	Creole	F	36	15	1:40	40.0	Positive	Respiratory, keratoconjunctivitis, corneal ulcer, progressive weight loss, evolution to prostration and myoclonus.	Died*.
39	Pit bull blue	F	60	22	1:320	40.3	Positive	Respiratory, runny nose and conjunctivitis, weight loss, keratosis, evolved to ataxia and myoclonus.	Recovered with neurological sequelae.
40	Rottweiler cross	M	24	25	1:160	40.0	Positive	Respiratory, digestive, melena, keratoconjunctivitis, evolved to myoclonus and seizures.	Died.

41	Creole	M	N/D	18	1:80	39.9	Positive	Digestive, cachexia, melena, corneal opacity, keratosis, evolved to ataxia and general myoclonus	Died.
42	Labrador	F	48	20	1:40	39.8	Positive	Digestive, weight loss, melena, conjunctivitis, keratosis, evolved to seizures.	Died.
43	Cross Boxer	M	84	24	1:80	40.5	Positive	Respiratory, keratoconjunctivitis, cachexia, plantar keratosis, evolved to seizures.	Died.
44	Chow Chow cross	M	96	22	1:40	40.1	Positive	Respiratory, nasal and plantar keratosis, corneal ulcer, weight loss, evolved to seizures.	Died.
45	Creole	F	18	20	1:160	39.9	Positive	Respiratory, nasal and ophthalmic discharge, corneal opacity, cachexia, evolved to myoclonus in limbs and parietals.	Died.
46	Pit Bull	F	72	18	1:160	40.2	Positive	Respiratory and digestive conjunctivitis with discharge, cachexia, melena, evolved to prostration and seizures.	Died.

M: Male, F: Female, Kg: Kilogram, IgM: Virus-specific immunoglobulin M/Value of immunoglobulin M, Indirect Immunofluorescent Assay (IFA) test was used for the quantification of the IgM, Temp.: Temperature, RT-PCR: Reverse Transcriptase Polymerase Chain Reaction, *Due to the general discomfort of the dog, the owner suspended the treatment and decided to euthanize.
