# Supplementary material for: Evaluation of the Efficacy and Safety of Silver Nanoparticles in the Treatment of Non-Neurological and Neurological Distemper in Dogs: A Randomized Clinical Trial
Source: Viruses. 2022 Oct 24;14(11):2329. doi: 10.3390/v14112329 (PMC9694365; doi:10.3390/v14112329)
Supplement: Supplementary file 1 [file viruses-14-02329-s001.zip › Table S4.pdf]

Table S4. Characteristics and results obtained for the dogs with neurological distemper treated with AgNPs (Group 2a).

No.	Breed	Sex	Age (months)	Weight (kg)	IgM Value	Temp. (ºC)	RT-PCR	Clinical signs	Report
1	Pit Bull	F	72	27	1:160	40.9	Positive	Neurological, respiratory, myoclonus and temporary conjunctivitis.	Recovered without sequelae.
2	Pit Bull	F	29	28	1:320	40.6	Positive	Neurological, respiratory, corneal opacity conjunctivitis and general myoclonus.	Recovered with neurological sequelae.
3	Poodle mix.	M	12	9	1:160	40.6	Positive	Neurological, digestive, melena diarrhea, conjunctivitis, keratosis and ataxia.	Recovered without sequelae.
4	Creole	M	84	20	1:80	41.1	Positive	Neurological, cachexic, ataxic, forelimb tremors and keratosis.	Recovered without sequelae.
5	Pit Bull	M	12	22	1:80	39.9	Positive	Neurological, respiratory, digestive, swollen glands, throat, conjunctivitis and melena diarrhea.	No recovered, Euthanasia.
6	Schnauzer	F	132	6.5	1:320	40.9	Positive	Neurological, prostration, myoclonus in limbs and parietals.	Recovered without sequelae.

7	Mixed	F	12	20	1:80	40.9	Positive	Neurological, respiratory, ataxia, keratoconjunctivitis, melena and myoclonus.	Recovered without sequelae.
8	Creole	M	84	22	1:80	39.7	Positive	Neurological, cachexia, conjunctivitis, tremors in limbs and convulsive.	Recovered without sequelae.
9	Husky	F	36	26	1:160	40.1	Positive	Neurological, tremors, ataxia, conjunctivitis and keratosis.	Recovered without sequelae.
10	Pug	F	60	6	1:80	40.2	Positive	Neurological, keratoconjunctivitis and seemed posterior.	No recovered, Euthanasia. *
11	Pug	M	48	6.5	1:160	39.7	Positive	Neurological, convulsive and myoclonus.	No recovered, Euthanasia. *
12	Creole	F	41	25	1:80	40.7	Positive	Neurological, keratosis, melena, ataxia and tic in the jaw.	Recovered with neurological sequelae.
13	Chihuahua Mix.	M	12	7	1:80	40.8	Positive	Neurological, myoclonus in the forelimbs and parietal.	No recovered, Euthanasia. *

14	Belgian Shepherd	F	48	16	1:80	39.7	Positive	Neurological, convulsive, keratosis, cachexia and general myoclonus.	Died.
15	Rottweiler	M	36	32	1:320	39.9	Positive	Neurological, Respiratory, keratoconjunctivitis, ataxia and parietal myoclonus.	Recovered without sequelae.
16	Pit Bull	M	24	31	1:80	41.1	Positive	Neurological, digestive, melena, runny nose, conjunctivitis, ataxia and seizures.	Died.
17	Chihuahua	F	36	4	1:160	40.9	Positive	Respiratory, neurological, conjunctivitis and melena, tic in the jaw and parietal.	Recovered without sequelae.
18	Poodle-Maltese	M	84	7.5	1:80	40.0	Positive	Respiratory, neurological, cachexia, conjunctivitis, runny nose, tics in the abdomen and hind limb.	Recovered without sequelae.
19	Creole	M	72	18	1:160	39.8	Positive	Neurological, digestive, vomiting, melena diarrhea, conjunctivitis, keratosis, ataxia and abdominal tic.	Recovered without sequelae.
20	York Shire	F	24	3.5	1:80	40.2	Positive	Neurological, respiratory, corneal opacity, keratosis and cachexia, ataxia and tremors.	Recovered with neurological sequelae.
21	Rottweiler	F	48	30	1:160	40.4	Positive	Respiratory, neurological, digestive, conjunctivitis, melena and tic in the forelimb.	Recovered without sequelae.
22	German	M	108	36	1:80	40.2	Positive	Neurological, cachexia, conjunctivitis, keratosis, ataxia, myoclonus and tremors.	Recovered

	Shepherd								without sequelae.
23	Schnauzer	F	72	8	1:320	39.8	Positive	Respiratory, neurological, conjunctivitis, cough and ataxia.	Recovered without sequelae.
24	GDP Bulldog	M	41	30	1:160	40.2	Positive	Neurological, respiratory, runny nose, temporal myoclonus and total prostration.	Recovered without sequelae.
25	Golden Retriever	F	24	24	1:80	39.9	Positive	Respiratory, digestive, melena-diarrhea, keratosis, conjunctivitis and ataxia.	Recovered with neurological sequelae.
26	Belgian Shepherd	F	24	22	1:160	40.2	Positive	Digestive, neurological, cachexia, diarrhea, keratosis and general myoclonus.	Recovered without sequelae.
27	Mixed Doberman	F	96	28	1:40	40.6	Positive	Neurological, respiratory, opaque corneal conjunctivitis, myoclonus and seizures.	Recovered with neurological sequelae.
28	Pug	M	13	6.5	1:80	39.9	Positive	Neurological and respiratory.	Recovered with neurological sequelae.

29	Creole	F	12	9	1:160	40.3	Positive	Respiratory, digestive, neurological, diarrhea, conjunctivitis, ataxia and tic in four limbs.	Died.
30	Boxer	M	9	15	1:160	40.6	Positive	Neurological, digestive, ataxia, melena and tic in the diaphragm.	Recovered without sequelae.
31	Chihuahua	F	72	3.5	1:80	39.6	Positive	Neurological respiratory, nasal and ophthalmic discharge, temporal tremors and tic in the jaw.	Recovered without sequelae.
32	Labrador	M	24	23	1:160	41.1	Positive	Digestive, neurological, cachexia, diarrhea, melena, ataxia and abdominal spasm.	Recovered without sequelae.
33	Cocker	F	120	12	1:160	39.9	Positive	Digestive, diarrhea, vomiting, neurological, keratoconjunctivitis, frontal claudication and ataxia.	Recovered with neurological sequelae.
34	Pit Bull	M	23	28	1:40	39.9	Positive	Respiratory corneal opacity. Neurological, tremors and abdominal tic.	Recovered without sequelae.
35	Poodle	F	36	8	1:160	40.2	Positive	Digestive, diarrhea, melena. Neurological, tic in the abdomen, tremors and general pain.	Recovered without sequelae.
36	Akita	M	60	38	1:160	40.7	Positive	Neurological, cachexic, ataxic, posterior prostration and keratosis.	Recovered without

									sequelae.
37	Mixed Dachshunds	M	24	5	1:80	40.5	Positive	Respiratory, neurological, keratoconjunctivitis, seizures and prostration.	Euthanasia.
38	Husky	F	48	23	1:320	39.9	Positive	Neurological, digestive, conjunctivitis and melena diarrhea, forelimb and parietal tic.	Recovered without sequelae.
39	German Shepherd	M	12	28	1:80	40.0	Positive	Neurological, respiratory, keratoconjunctivitis and diaphragm and temporal tics.	Recovered without sequelae.
40	Shiatzu	M	60	4	1:160	39.8	Positive	Neurological, keratoconjunctivitis and ataxia and parietal tremor.	Recovered with neurological sequelae.
41	Creole	F	36	17	1:40	40.3	Positive	Neurological, conjunctivitis, keratosis, ataxia and parietal tic and abdominal spasm.	Died.
42	Golden	M	72	22	1:160	40.5	Positive	Respiratory, digestive, melena, conjunctivitis and keratosis, abdominal spasm.	Recovered without sequelae.
43	Creole	F	0.7	22	1:160	40.2	Positive	Respiratory, nasal and ophthalmic discharge, conjunctivitis and keratosis, seizures.	Died.
44	Mixed	M	24	7	1:80	39.0	Positive	Neurological, ataxia, keratoconjunctivitis, melena and myoclonus.	Died.
45	Chihuahua	M	24	2.5	1:40	40.1	Positive	Neurological, conjunctivitis, melena and keratosis.	Recovered without sequelae.

46	Pit bull	M	24	24	1:320	39.8	Positive	Respiratory, digestive, diarrhea melena conjunctivitis and evolved to seizures.	Died.
47	Husky	M	72	26	1:160	40.9	Positive	Respiratory, conjunctivitis, keratosis, melena. It progressed to ataxia and seizures.	Died.
48	Chihuahua	F	108	4.5	1:40	39.9	Positive	Respiratory, runny nose, cachexia, conjunctivitis, evolved to complete prostration and seizures.	Euthanasia.
49	Boston	M	96	19	1:80	40.	Positive	Respiratory, sneezing, cachexia, nasal discharge, corneal opacity and conjunctivitis, myoclonus in extremities.	Recovered with neurological sequelae.
50	Pug	M	48	6	1:160	40.0	Positive	Respiratory, keratoconjunctivitis, corneal opacity and cachexia, parietal and maxillary myoclonus.	Died.
51	Labrador	M	36	25	1:80	40.	Positive	Respiratory, conjunctivitis, sneezing, secretions, cachexia and melena.	Euthanasia.
52	Boxer	F	48	22	1:160	39.7	Positive	Respiratory, digestive, melena, nasal and ophthalmic secretions, cachexia.	Recovered without sequelae.
53	Schnauzer	M	72	9	1:320	39.9	Positive	Digestive, keratoconjunctivitis, bloody diarrhea and cachexia, prostration and seizures.	Recovered without sequelae.
54	Chihuahua-Pug	M	12	5	1:80	40.6	Positive	Respiratory, keratoconjunctivitis, corneal opacity and cachexia, evolved to seizures.	Recovered with neurological sequelae.
55	Pit Bull	F	4	7	1:80	39.1	Positive	Neurological, conjunctivitis and melena diarrhea.	Died.
56	Chihuahua	M	108	3.5	1:160	40.0	Positive	Digestive, respiratory, cough, melena diarrhea, conjunctivitis, evolved to seizures.	Died.

57	Boxer-Pit Bull Mix	M	24	20	1:80	40.1	Positive	Respiratory, conjunctivitis, cachexia, seizures, keratosis and opaque corneas.	Euthanasia.
58	Chihuahua	F	108	4	1:160	40.1	Positive	Neurological, keratosis, loss of the swallowing reflex and seizures.	Died.

M: Male, F: Female, Kg: Kilogram, IgM: Virus-specific immunoglobulin M/Value of immunoglobulin M, Indirect Immunofluorescent Assay (IFA) test was used for the quantification of the IgM, Temp.: Temperature, RT-PCR: Reverse Transcriptase Polymerase Chain Reaction, *Due to the general discomfort of the dog, the owner suspended the treatment and decided to euthanize.
