# Supplementary material for: Evaluation of the Efficacy and Safety of Silver Nanoparticles in the Treatment of Non-Neurological and Neurological Distemper in Dogs: A Randomized Clinical Trial
Source: Viruses. 2022 Oct 24;14(11):2329. doi: 10.3390/v14112329 (PMC9694365; doi:10.3390/v14112329)
Supplement: Supplementary file 1 [file viruses-14-02329-s001.zip › Table S5.pdf]

Table S5. Characteristics and results obtained for the dogs with neurological distemper treated with only supportive therapy (Group 2b).

No.	Breed	Sex	Age (months)	Weight (kg)	IgM Value	Temp. (ºC)	RT-PCR	Clinical signs	Report
1	Pit Bull	F	84	28	1:40	40.1	Positive	Temporal myoclonus, keratoconjunctivitis and tic in the maxilla.	Died.
2	Pug	F	24	4	1:160	40.3	Positive	Neurological, keratoconjunctivitis, seemed posterior and slight seizures.	Died.
3	Chihuahua Mix	F	48	3.5	1:80	40.6	Positive	Neurological, conjunctivitis, corneal opacity, keratosis, melena, ataxia and seizures.	Died.
4	Husky	M	24	24	1:80	39.6	Positive	Keratoconjunctivitis, ataxia, progressed to myoclonus and seizures.	Euthanasia *.
5	German Shepherd	M	26	30	1:160	41.1	Positive	Keratoconjunctivitis, cachexia, melena, and seizures.	Euthanasia *.
6	Mixed Dalmatian	F	27	22	1:80	39.7	Positive	Runny nose, cachexia, keratoconjunctivitis and myoclonus legs.	Died.
7	Creole	M	12	21	1:80	40.5	Positive	Cachexia, diarrhea, abdominal spasm, and general myoclonus.	Died.
8	Boxer Cross - Pit Bull	F	36	18	1:160	40.3	Positive	Keratoconjunctivitis, melena, myoclonus, parietal and maxillary tics.	Euthanasia *.
9	Pit Bull	M	60	25	1:160	39.9	Positive	Seizures, keratoconjunctivitis, parietal myoclonus and left forelimb.	Died.
10	Chihuahua	F	72	3.6	1:320	40.4	Positive	Keratoconjunctivitis, prostration	Died.

								and myoclonus and seizures.	
11	Creole	M	9	12	1:40	39.8	Positive	Tremors, parietal myoclonus and maxillary tic.	Died.
12	Creole	F	10	11	1:80	40.3	Positive	Ataxia, conjunctivitis with discharge, parietal myoclonus, and seizures.	Died.
13	Belgian Shepherd	F	24	23	1:160	41.0	Positive	Keratoconjunctivitis, diarrhea, ataxia, and seizures.	Died.
14	Creole	F	24	12	1:40	38.7	Positive	Keratoconjunctivitis, cachexia, melena, and seizures.	Died.
15	Husky	F	60	26	1:40	39.6	Positive	Keratoconjunctivitis, melena, ataxia and myoclonus in limbs.	Euthanasia*.
16	German Shepherd	F	48	27	1:160	41.7	Positive	Neurological, keratoconjunctivitis, ataxia with evolved to seizures.	Died.
17	Poodle	M	24	5	1:320	41.0	Positive	Prostration, abdominal spasms and seizures.	Died.
18	Schnauzer	M	72	8	1:80	40.5	Positive	Temporal myoclonus and legs, evolved to seizures.	Euthanasia*.
19	Creole	M	36	8	1:80	40.1	Positive	Ataxia, diarrhea, conjunctivitis, evolved to seizures.	Died.
20	Chihuahua	F	84	4	1:160	39.9	Positive	Nasal and plantar keratosis, prostration and seizures.	Euthanasia*.
21	Rottweiler	F	48	25	1:320	41.2	Positive	Ataxia, abdominal spasm, progressed to prostration and seizures.	Died.
22	Maltese	M	96	7	1:40	40.2	Positive	Keratoconjunctivitis, lower jaw tic, swallowing reflex paralysis.	Died.

23	Dachshund	M	36	6.3	1:320	39.9	Positive	Cachexia, conjunctivitis, general myoclonus.	Died.
24	Creole	F	60	5.5	1:80	40.1	Positive	Ataxia, conjunctivitis with discharge, parietal myoclonus, seizures.	Died.
25	Boxer	M	36	23	1:40	39.8	Positive	Prostration, tics in limbs and convulsions.	Died.
26	Rottweiler	M	24	24	1:40	40.5	Positive	Prostration, tic in the jaw, keratoconjunctivitis melena.	Euthanasia *.
27	Belgian Shepherd.	F	96	21	1:160	40.2	Positive	Ataxia, evolved to abdominal spasm and seizures.	Died.
28	Australian pastor	M	48	18	1:40	39.7	Positive	Myoclonus in extremities, abdominal spasm, later prostration.	Euthanasia *.
29	Maltese-Poodle	F	120	8	1:80	39.6	Positive	Conjunctivitis, ataxia, progression to general myoclonus, and prostration.	Died.
30	Schnauzer	M	48	7	1:320	39.9	Positive	Conjunctivitis, cranial limb tics, seizures.	Euthanasia *.
31	Doberman	M	24	25	1:160	40.0	Positive	Keratoconjunctivitis, abdominal spasm, jaw and parietal tics.	Died.
32	Yorkshire	F	96	3.4	1:40	41.2	Positive	Seizures, ataxia, cachexia, and conjunctivitis.	Died.
33	Golden retriever	M	72	22	1:40	40.2	Positive	Cachexia, melena, conjunctivitis, subsequent prostration and coma.	Died.
34	Creole	F	60	17	1:320	39.6	Positive	Cachexia, abdominal spasm, convulsive.	Died.
35	Creole	F	12	23	1:80	39.8	Positive	Tics in the extremities and evolved to seizures.	Died.

36	Pit Bull	F	12	22	1:40	40.5	Positive	Keratoconjunctivitis, nervous tics in extremities, melena and cachexia.	Died.
37	Chihuahua	M	120	4.4	1:160	40.0	Positive	Convulsive, ataxia, conjunctivitis, melena.	Died.
38	German shepherd	F	96	26	1:40	39.7	Positive	Cachexia, melena, hindquarters and seizures.	Euthanasia *.
39	Pug	M	36	7	1:80	40.4	Positive	Cachexia, runny nose and eye, seizures.	Died.
40	Chihuahua	M	60	3.5	1:40	39.9	Positive	Conjunctivitis, ataxia, later limb tics and prostration.	Euthanasia *.
41	Cocker	F	24	8	1:40	40.1	Positive	Conjunctivitis with discharge, cachexia, ataxia, seizures.	Euthanasia *.
42	Shiatzu	F	48	5	1:80	39.8	Positive	Cachexia, melena, general myoclonus and seizures,	Died.
43	Akita	F	72	35	1:40	40.3	Positive	Nasal and plantar keratosis, cachexia, mandibular tics and cranial extremities.	Died.
44	Creole	M	18	21	1:80	41.1	Positive	Cachexia, melena, abdominal spasm, mandible and parietal myoclonus.	Died.
45	Sharpei cross	M	36	20	1:160	40.1	Positive	Conjunctivitis, keratosis, cachexia, melena, and seizures.	Died.
46	Belgian Shepherd	F	36	22	1:40	39.9	Positive	General myoclonus and seizures.	Euthanasia *.
47	Bull terrier	M	84	15	1:320	40.8	Positive	Conjunctivitis, cachexia, ataxia, and seizures.	Euthanasia *.
48	Old English Shepherd	M	108	24	1:40	40.3	Positive	Convulsive, cachexia, melena and keratosis.	Died.
49	Creole	F	n/d	12	1:80	40.4	Positive	Cachexia, ataxia, myoclonus in the	Died.

								caudal extremities and final prostration.	
50	Doberman	F	36	24	1:160	39.8	Positive	Keratosis, conjunctivitis, parietal myoclonus and mandible.	Died.
51	Boxer cross	M	108	19	1:40	40.0	Positive	Myoclonus in extremities, abdominal spasm and seizures.	Euthanasia *.

M: Male, F: Female, Kg: Kilogram, IgM: Virus-specific immunoglobulin M/Value of immunoglobulin M, Indirect Immunofluorescent Assay (IFA) test was used for the quantification of the IgM, Temp.: Temperature, RT-PCR: Reverse Transcriptase Polymerase Chain Reaction, *Due to the general discomfort of the dog, the owner suspended the treatment and decided to euthanize.
